# Identification of Antimicrobial Peptide Genes in Black Rockfish *Sebastes schlegelii* and Their Responsive Mechanisms to *Edwardsiella tarda* Infection

**DOI:** 10.3390/biology10101015

**Published:** 2021-10-09

**Authors:** Min Zhang, Min Cao, Yunji Xiu, Qiang Fu, Ning Yang, Baofeng Su, Chao Li

**Affiliations:** 1 School of Marine Science and Engineering, Qingdao Agricultural University, Qingdao 266109, China; zhangmin@qau.edu.cn (M.Z.); caomin@qau.edu.cn (M.C.); yunji16@qau.edu.cn (Y.X.); qiangfu@qau.edu.cn (Q.F.); yangning@qau.edu.cn (N.Y.); 2 School of Fisheries, Aquaculture and Aquatic Sciences, Auburn University, Auburn, AL 36849, USA; BZS0014@auburn.edu

**Keywords:** *Sebastes schlegelii*, genome sequencing, antimicrobial peptide genes (AMPs), *Edwardsiella tarda*, gene expression

## Abstract

**Simple Summary:**

*Sebastes schlegelii* is a typical viviparous teleost that belongs to the family Scorpaenidae, and is an important mariculture fish in East Asian countries such as Japan, Korea, and China because of its high economic and ecological values. With the enlargement of cultivation scale, bacterial and viral diseases have become the main threats to the farming industry of *S. schlegelii*, which resulted in significant economic losses. The genome features and evolutionary position of *S. schlegelii* were investigated based on multiple sequencing approaches. Then, genome-wide identification of AMPs was performed according to the known AMPs in the Antimicrobial Peptides Database. Furthermore, the expression levels of these AMPs were detected to explore their responsive mechanisms to pathogenic bacteria. The availability of the high-quality genome of *S. schlegelii* will be valuable for understanding its genome features and evolutionary history. Genome-wide AMP identification could be beneficial for understanding the molecular mechanism of innate and adaptive immune responses of *S. schlegelii*.

**Abstract:**

The black rockfish, *Sebastes schlegelii*, is a typical viviparous teleost, which belongs to the family Scorpaenidae. Due to its high economic and ecological values, *S. schlegelii* has been widely cultured in East Asian countries. With the enlargement of cultivation scale, bacterial and viral diseases have become the main threats to the farming industry of *S. schlegelii*, which have resulted in significant economic losses. In this study, Illumina shotgun sequencing, single-molecule real-time (SMRT) sequencing, 10× genomics and high-throughput chromosome conformation capture (Hi-C) technologies were collectively applied to assemble the genome of *S. schlegelii*. Then, we identified the antimicrobial peptide genes (AMPs) in the *S. schlegelii* genome. In total, 214 AMPs were identified in the *S. schlegelii* genome, which can be divided into 33 classes according to the annotation and cataloging of the Antimicrobial Peptides Database (APD3). Among these AMPs, thrombin-derived C-terminal peptide (TCP) was the dominant type, followed by RegIIIgamma and chemokine. The amino acid sequences of the TCP, cgUbiquitin, RegIIIalpha, RegIIIgamma, chemokine shared 32.55%, 42.63%, 29.87%, 28.09%, and 32.15% similarities among the same type in *S. schlegelii.* Meanwhile, the expression patterns of these AMPs in nine healthy tissues and at different infection time points in intestine were investigated. The results showed that the numbers and types of AMPs that responded to *Edwardsiella tarda* infection gradually increased as the infection progressed. In addition, we analyzed the phylogenetic relationships of hepcidins in teleost. The identification of AMPs based on the whole genome could provide a comprehensive database of potential AMPs, and benefit for the understanding of the molecular mechanisms of immune responses to *E. tarda* infection in *S. schlegelii*. This would further offer insights into an accurate and effective design and development of AMP for aquaculture therapy in the future.

## 1. Introduction

Black rockfish (*Sebastes schlegelii*), a typical viviparous teleost, belongs to the family Scorpaenidae [1]. It is an important mariculture fish in East Asian countries such as Japan, Korea, and China because of its high economic and ecological values [2]. Previous study showed that *S. schlegelii* contains high nutrient contents, such as proteins, unsaturated fatty acids, and amino acids, among others, which are beneficial for human beings [3]. Meanwhile, it has been found that characteristics such as disease resistance, tolerance to low water temperature, and fast growth rate make it suitable for large-scale aquaculture [4]. Usually, *S. schlegelii* inhabits in coastal rocky reefs, feeds on benthic animals, and can survive in temperatures ranging from 5 °C to 28 °C, with the optimal temperature ranging from 18 °C to 24 °C [5]. The living environment makes *S. schlegelii* possess good tolerance to low water temperature. It has also been found that *S. schlegelii* can be selected as a model for reproduction research due to its viviparous reproduction method [6]. Therefore, these characteristics make *S. schlegelii* a promising fish species for coastal fisheries, and a good model for studying the molecular mechanism of environmental tolerance and developmental process.

Researchers aimed to increase the efficient production and nutrition components of *S. schlegelii* through optimizing their feeding schedules [7,8,9]. However, various factors, such as metal, ascorbic acid, ammonia, and temperature, are other influencing factors for the farming industry of *S. schlegelii*, which can impact economic gain in its aquaculture [10,11,12]. Therefore, the present studies were also conducted to aid in the development of disease prevention and control measures [13,14]. With the development of molecular biology, knowledge about the immunologic function of *S. schlegelii* becomes crucial for understanding the molecular mechanism of disease resistance. For example, the characterization of several immune-related genes in *S. schlegelii* have been performed, including PGRP, LTLs, KSPI, CTL, mTOR, MyD88, CCL25, cystatin B, and some PRRs such as TLRs and NLRs [15,16,17,18,19,20,21,22,23,24] to better understand the cellular and molecular responses to various stresses. In addition, several non-coding RNAs such as miRNA [25] and cricRNA [26] were also identified to explain their regulatory functions in immunity in black rockfish.

The aquaculture industry has grown dramatically and plays an important role in the world’s food security. However, aquaculture production is plagued by a range of different diseases and parasites. Vibriosis is among the most common diseases, which can lead to massive mortality in aquaculture species. For example, *Vibrio* spp. can cause viral encephalopathy and retinopathy, which is also associated with enteropathic larval syndrome in gilthead sea bream [27]. Mycobacteriosis has been reported in many fish species worldwide, which can cause high mortality among farmed pikeperch with a 0.2%/d rate [28]. In addition, it has been demonstrated that *Aeromonas salmonicida* can cause furunculosis that was correlated by high mortality and morbidity [29]. *Edwardsiella tarda* is a Gram-negative pathogen with a broad range of the host, and can cause bacterial diseases, known as Edwardsiellosis, resulting in massive economic losses [30]. To solve disease infection problems in aquaculture, various approaches such as vaccination, improvement in culture systems, and antibiotics have been developed. Immunostimulants are another developed method for application in the green and sustainable development of aquaculture. Therefore, it is necessary to understand aquatic animals’ immunologic mechanisms. 

The innate and adaptive immunity constitute the immune system of vertebrates [31]. Antimicrobial peptides (AMPs), a kind of small peptide molecules with positively charged ions (18–46 amino acids), showed broad spectrum antimicrobial activities against microorganisms such as bacteria, viruses, and fungi, and had immunomodulatory functions [32]. AMPs are conserved in their functions with hydrophilic sides, which can help it bind to microbial membrane to kill the microorganisms [33]. During the process of micro-organisms invasion, AMPs act as bridge between innate and adaptive immunity, and enhance the host ability to fight against microbial infections by activating the adaptive immune system [34]. To date, thousands of antimicrobial peptides have been characterized. It has been reported that fish are a great source of AMPs, including defensins, cathelicidins, hepcidins, histone-derived peptides, and fish specific-ones [35]. For example, different copies of fish defensins have been identified in zebrafish (*Danio rerio*), fugu (*Takifugu rubripes*), tetraodon (*Tetraodon nigroviridis*), gilthead seabream (*Sparus aurata*), Atlantic cod (*Gadus morhua*), and Nile tilapia (*Oreochromis niloticus*) [36,37,38,39,40]. Similarly, varying numbers of cathelicidin genes have been identified in different fish species [41]. Meanwhile, piscidin and piscidin family member genes have been identified in a number of fish species including seabass (*Dicentrarchus labrax*) [42], icefish (*Chionodraco hamatus*) [43], rock bream (*Oplegnathus fasciatus*) [44], large yellow croaker (*Larimichthys crocea*) [45], catfish (*Heteropneustes fossilis*) [46], and African butterfly fish (*Pantodon buchholzi*) [47]. Moreover, multi-copies of hepcidin were found in fish due to the whole genome duplication event, which is different from that in humans. To date, it has been demonstrated that more than dozens of fish harbor hepcidins [35]. It has been demonstrated that fish AMPs can be induced by pathogens and have direct broad-spectrum antimicrobial activity towards various pathogens. For example, pathogen challenge usually resulted in the rapid induction of β-defensin, cathelicidin, and piscidin at transcriptomic levels [48,49]. Moreover, the hepcidins of fish possess antimicrobial activity towards Gram-negative bacteria, Gram-positive bacteria, viruses, and parasites [41]. In *S. schlegelii*, however, only few AMPs have yet been reported. For example, Kim et al. identified and analyzed two hepcidin genes from *S. schlegelii* [50]. Therefore, a systematic identification and characterization of AMPs in *S. schlegelii* is still necessary.

In this study, Illumina shotgun sequencing, single molecule real-time (SMRT) sequencing, 10× genomics and high-throughput chromosome conformation capture (Hi-C) techniques were collectively employed to assemble the genome of *S. schlegelii*. The genome features and evolutionary position were further investigated. A genome-wide identification of AMPs was performed according to the known AMPs in Antimicrobial Peptides Database. Meanwhile, the expression levels of these AMPs were determined to explore their response mechanisms to pathogenic bacteria. Additionally, the comparison of these AMPs was analyzed in this study. The availability of the high-quality genome of *S. schlegelii* will be valuable for understanding its genome features and evolutionary history. Meanwhile, genome-wide AMPs identification could benefit the understanding of the molecular mechanism of innate and adaptive immune responses of *S. schlegelii*.

## 2. Materials and Methods

### 2.1. Samples Collection

Experimental adult *S. schlegelii* were collected from Qingdao, Shandong Province. Samples for DNA extraction were immediately frozen at −80 °C for the following use. The genomic DNA of *S. schlegelii* was isolated using Tissue DNA kit (Qiagen) according to the manufacturer’s instructions. High-quality DNA was used for both Illumina and PacBio libraries construction. RNA was extracted from *S. schlegelii* using TRIzol Reagent (Invitrogen, Carlsbad, CA, USA), followed by RNase-Free DNase I treatment to remove any DNA (TIANGEN, Beijing, China) according to the manufacturer’s instructions. The quantity and integrity of the genomic DNA were measured using NanoPhotometer^®^ spectrophotometer and Qubit^®^ DNA Assay Kit and agarose gel electrophoresis, respectively. The results showed A260/A280 and A230/A260 were all ≥1.8, which met the following sequencing requirements. Finally, DNA and RNAs were used for the following library construction and sequencing. 

### 2.2. Libraries Construction and Sequencing

High-quality genomic DNA was used to construct Illumina paired-end sequencing library with 350 bp insert sizes according to the manufacturer’s instructions provided by Illumina (San Diego, CA, USA). Meanwhile, a 20 kb library was constructed using the PacBio SMRTbell template kit 1.0 (Pacific Biosciences, Menlo Park, CA, USA) according to the manufacturer’s instructions after the size selection using BluePippen system (Sage Science, Inc., Beverly, MA, USA). Subsequently, the fragmented DNA was purified using AMPure XP beads (Beckman Coulter). Additionally, a 10× Genomics library was constructed and sequenced with paired-end 150 bp on the Illumina Hiseq platform. For the Hi-C library construction, chromatin in the nucleus was fixed in aldehyde, and then the fixed chromatin was digested with MboI to an average length of ~500 bp. The enzyme digested ends were biotinylated and ligated to form chimeric junctions. Subsequently, the Hi-C library was size-selected for 300–500 bp fragments and also sequenced on the Illumina HiSeq platform. Before assembly, adaptor sequences and low-quality reads from Illumina HiSeq and PacBio platforms were removed. The sequencing data have been deposited into the NCBI SequenceRead Archive with the Bioproject Number: PRJNA516036.

### 2.3. Genome Assembly and Genome Evaluation 

We assembled the *S. schlegelii* genome using a combination of shotgun reads, long-read SMRT sequencing, 10× Genomics, and Hi-C maps. The 17-mer frequency was calculated by Jellyfish and genome size was estimated by the formula G = N_17-mer_/D_17-mer_ [51] based on the shotgun reads generated from the Illumina platform. The *S. schlegelii* genome was assembled using long reads after self-correction from PacBio RSII platform using the RS_HGAP_Assembly.3 protocol in SMRT Analysisv2.3.0 [52]. Quiver was run to polish the accurate sequences. Subsequently, data from 10× Genomics library was used to produce scaffold using fragscaff [53]. For further scaffolding construction, reads from Hi-C were mapped onto the genome assembly using BWA software (v0.7.16, http://bio-bwa.sourceforge.net/, accessed date: 18 October 2018) with parameters (-n 0) to avoid mismatches and non-specific alignments in repetitive and homoeologous regions. Contigs were then ordered and oriented using the 3d-DNA pipeline with default parameters. After assembly, the quality and integrity of the genome was assessed through the following three approaches. Short reads were mapped onto the assembled genome using BWA software (http://bio-bwa.sourceforge.net/, parameters ‘-o 1 -i 15′, accessed date: 28 October 2018). The Core Eukaryotic Genes Mapping Approach (CEGMA) can help build an initial set of reliable gene annotations and ensure the reliability of the gene structures [54]. To further evaluate the completeness of the *S. schlegelii* genome assembly, Benchmarking Universal Single-Copy Orthologs (BUSCO) was used to benchmark the completeness of *S. schlegelii* genome [55]. 

### 2.4. Gene Prediction and Annotation

De novo and homology-based methods were used to predict interspersed repeats in the *S. schlegelii* genome. RepeatModeler (version: 1.0.8) was used to analyze consensus sequences longer than 80 bp in genomes of *S. schlegelii*. Then, these consensus sequences were used as the library in RepeatMasker (version: open-4-0-7) to predict interspersed repeat elements in the whole genome. For the homology prediction, interspersed repeats were searched based on a repetitive sequence database (RepBase, http://www.girinst.org/repbase/, accessed date: 15 November 2018).) using the RepeatMasker and repeatproteinmask (http://www.repeatmasker.org/, accessed date: 15 November 2018). Additionally, Tandem Repeats Finder was used to identify tandem repeat sequences in *S. schlegelii* genome.

Subsequently, we used a combination of *de novo* prediction, homology searches and RNA-evidenced methods to predict gene structures of *S. schlegelii*. De novo predictions were performed using Augustus, GlimmerHMM, SNAP, Geneid and Genscan. Meanwhile, transcriptome of *S. schlegelii* were mapped to the genome using BLAST. Finally, EVidenceModeler (EVM) and PASA were used to integrate a non-redundant and complete gene set based on the gene models from the above three methods [56].

For the gene annotation of the protein-coding genes in *S. schlegelii*, genes were aligned to NR and Swiss-Prot protein databases using BLAST with an e-value of 1 × 10^−5^. Meanwhile, the motifs and conserved domains were scanned with InterProScan and searched against Pfam database. Furthermore, Blast2GO software was used to identify Go terms. Then, these protein-coding genes were searched against to the KEGG database (http://www.genome.jp/kegg, accessed date: 3 January 2019) to perform KEGG annotations for the whole genome. 

### 2.5. Genome Comparison

To estimate the gene family expansion and contraction, the genome of S. schlegelii combined with Cynoglossus semilaevis, Cyprinus carpio, Danio rerio, Gambusia affinis, Ictalurus punctatus, Larimichthys crocea, Lateolabrax maculates, Latimeria chalumnae, Oryzias latipes, Paralichthys olivaceus, Poecilia latipinna, Poecilia reticulate, Xiphophorus maculates and Mus musculus were selected to define orthologous genes using orthoMCL and CAFE [57,58]. 

### 2.6. Phylogenetic Tree Reconstruction and Divergence Time Estimation

In order to illuminate the phylogenetic position of *S. schlegelii*, 572 single-copy orthologous genes among *S. schlegelii* and other 14 species were used in phylogenetic analyses. These genes were aligned using MUSCLE one by one and then formed a super alignment matrix under the default parameters [59]. Then, RAxML (v 8.2.12) was used to construct phylogenetic tree with default parameters [60]. Meanwhile, we calculated the divergence time of *S. schlegelii* using this phylogenetic tree based on seven time calibrations—*L. crocea* and *L. japonicas*(83~108 Ma), *X. maculates* and *G. affinis* (15.05~18.87 Ma), *L. japonicas* and *X. maculatus* (105~154 Ma), *D. rerio* and *C. carpio* (87.8~124.7 Ma), *D. rerio* and *I. punctatus* (126~179 Ma), *X. maculates* and *I. punctatus* (204.5~255.3), *D. rerio* and *M. musculus* (425~446 Ma)—using the mcmctree in the PAML package (http://abacus.gene.ucl.ac.uk/software/paml.html, accessed date: 10 March 2019). The parameters of timetree were set as follows: burn-in = 5,000,000; sample number = 1,000,000; sample-frequency = 50. 

### 2.7. Identification and Analysis of AMPs

For the AMPs identification, we downloaded 2927 known AMP sequences from Antimicrobial Peptides Database (APD3, http://aps.unmc.edu/AP/main.php, accessed date: 7 August 2019) and built a database for the following analysis. Then, we queried the proteins of *S. schlegelii* against the above database with the e-value as 1 × 10^−5^. We performed classification of these AMPs according to the classical peptides. Meanwhile, we predicted AMP genes of *I. punctatus*, *C. carpio*, *D. rerio*, *O. latipes*, *P. reticulata*, *P. latipinna*, *M. albus, P. olivaceus, C. semilaevis*, *L. crocea*, and *L. chalumnae* based on their genome using the same method mentioned above, and then performed a comparison among *S. schlegelii* and these species. For AMPs in *S. schlegelii*, the top-five types of AMP genes from *S. schlegel* were aligned to detect sequences diversity. 

### 2.8. Healthy Tissues Extraction and Bacterial Challenge 

To explore the expression levels of these predicted AMP genes in *S. schlegelii*, individuals (average body length: ~15 cm) were cultured in a flow-through system and acclimatized for 1 week at 28 °C before experiment. For healthy tissues, intestine, kidney, spleen, liver, brain, gill, muscle, brain, and blood were collected from healthy *S. schlegelii* for RNA extraction. For bacterial challenge, *E. tarda* was first isolated from a single colony and then was cultured in LB medium at 28 °C overnight with 180 rpm/min. Before infection, we plated 100 μL of 10-fold serial dilutions bacteria onto plates and then calculated their concentration using colony forming units (CFUs) per mL. Subsequently, the fish in experimental groups were immersed in 30 L aquaria with a final concentration of 1 × 10^7^ CFU/mL of *E. tarda* for 4 h [61]. In contrast, the fish in control groups (CON) were immersed in seawater. After 4 h immersion, the fish were transferred back in normal culture conditions. Subsequently, samples from control groups and *E. tarda* infected groups were separately collected at 2 h (EI2H), 6 h (EI6H), 12 h (EI12H), and 24 h (EI24H) post-challenge. The intestine tissues were collected from these fish per group after infection for the following use. Nine fish were used for sample collection for each tissue and each time point, and each replicate includes three random individuals. The tissues were frozen in liquid nitrogen, and then used for RNA extraction and cDNA synthesis as described above.

### 2.9. Gene Expression Analysis of AMP Genes

To analyze the expression patterns of AMP genes in *S. schlegelii*, we extracted RNA using the same method mentioned above. The integrity of the RNA was monitored on 1% agarose gels. RNA concentration was checked using a NanoPhotometer spectrophotometer (IMPLEN Inc, Westlake Village, CA, USA). For transcriptome library construction and sequencing, a total of 5 μg of RNA per sample was used to generate sequencing libraries with an Illumina TruSeq RNA Sample Preparation Kit (Illumina, San Diego, CA, USA) according to the manufacturer’s recommendations. Then, the prepared libraries were sequenced on an Illumina Hiseq 2000 platform. Thereafter, clean data of these libraries were obtained by removing low-quality reads, adapter sequences, and reads containing N, then were used for the downstream analysis. After quality filtering, the RNA sequencing reads from these tissues were aligned to the assembly genome using TopHat (v2.0.12). The RPKM values and expression patterns of these AMP genes were analyzed. RPKM values from blood of CON were selected as control. After the expression data of AMP genes were obtained, pheatmap program (version 1.0.12) in the R pakage was employed to generate the expressions.

### 2.10. Analysis of Hepcidins in Teleost

Hepcidin displays a broad spectrum of antibiotic activity against a variety of bacteria, viruses, fungi, and protozoa, being crucial in the innate immune response. Therefore, hepcidins from some available species were extracted to construct a phylogenetic tree using RAxML (v 8.2.12) with default parameters [60]. Hepcidins from *Homo sapiens* and *Mus musculus* were selected as outgroups. In addition, DNAMAN was used to exhibited similarities of hepcidins from *S. schlegelii*, *I. punctatus*, *C. carpio*, *D. rerio*, *O. latipes*, *P. reticulata*, *P. latipinna*, *M. albus, P. olivaceus, C. semilaevis*, *L. crocea*, and *L. chalumna*.

## 3. Results

### 3.1. Genome Sequencing and Assembly

**Figure 1:**
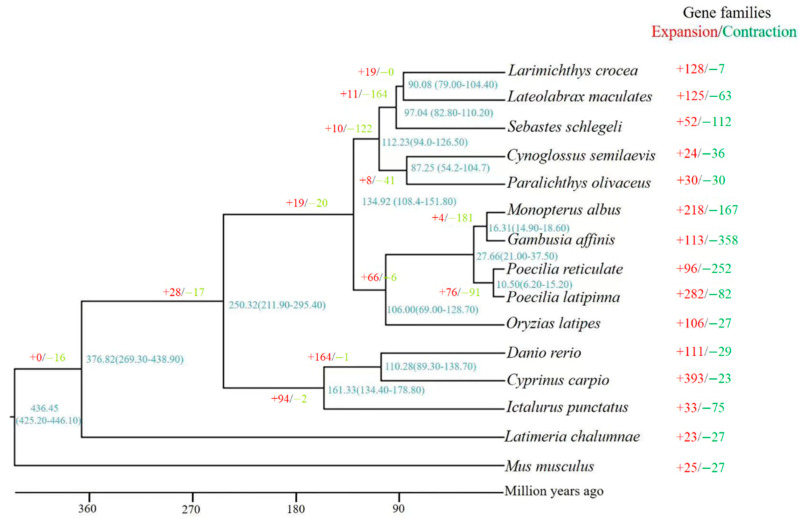
Phylogenetic relationships of *Sebastes schlegelii* and other species.

For the sequencing depth assessment, the 350 bp paired-end library generated ~102.30 Gb data with 121.32× sequencing depth of the S. schlegelii genome (assuming a genome size of 843.18 Mb). Meanwhile, ~84.18 Gb of data was obtained from PacBio platform (99.83× sequencing depth of the whole genome). Additionally, the total sequencing data of 10× Genomics library was ~120.94 Gb (143.43× sequencing depth of the whole genome). For the Hi-C library, a total of ~108.69 Gb data with 128.90× sequencing depth was generated. The statistical results of all data are presented in Table S1. Data from these four libraries were used to assemble the *S. schlegelii* genome. The estimated genome size of S. schlegelii based on 17 k-mer analysis is about 856.02 Mb (Figure S1). For scaffolding construction using long reads, the result showed the genome size of *S. schlegelii* was 844.74 Mb with contig N50 of 5.47 Mb (Table 1). For the scaffolding construction, reads from Hi-C were mapped onto the genome assembly. Finally, de novo assembly using combined techniques yielded an 848.87 Mb *S. schlegelii* genome with scaffold N50 of 35.73 Mb (Table 1). The average GC content of the whole genome is 40.93%. Based on chromatin interaction data, 97.42% of the assembled sequences were anchored onto 24 pseudo-chromosomes ranging from 1.70 Mb to 8.15 Mb in length (Table S2, Figure 1).

For the completeness assessment of the whole genome, the results showed that 98.99% of the short reads could map onto the assembled genome with a coverage of 99.05%, indicating the high accuracy of the assembly. Analysis of CEGMA revealed that the *S. schlegelii* genome harbored 228 complete and 10 partial genes, which occupied 96.37% of the eukaryotic core gene sets. The results of BUSCO showed that 96.01% (978 single-copy genes) of the eukaryotic single-copy genes were detected in this genome and the total proportions of fragment and missing genes accounted for only 3.9%, indicating the high integrity of the assembly.

### 3.2. Genome Prediction and Annotation

For the de novo and homology prediction of the repeats elements, the results showed that the repeat elements constituted 38.95% of the *S. schlegelii* genome, including 35.97% interspersed repeat and 2.80% tandem repeat sequences (Table S3). Repeat libraries were then used for repeats masking. For genome prediction, de novo predictions predicted 23,942 gene models for *S. schlegelii*. Based on this homologous protein database, 22,155 protein-coding sequences were obtained after removing redundancies. We predicted 18,904 gene models based on RNA evidence (Figure S2). Finally, EVidenceModeler (EVM) and PASA were used to integrate a non-redundant and complete gene set, and 24,134 gene models were identified. The transcript, CDS, exon and intron exhibited average lengths of 15116.74, 1612.07, 171.21 and 1604.67 bp, respectively.

For the gene annotation of the protein-coding genes in *S. schlegelii*, 99.05% of the protein-coding genes have been assigned at least one functional term. Among these, 95.30%, 89.60%, 82.20%, 99.20%, 92.80%, and 81.50% of *S. schlegelii* protein-coding genes showed homologies to Nr and Swiss-Prot protein, KEGG, InterPro, Go, and Pfam databases, respectively (Table S4). For the noncoding RNA annotation, 1671 miRNAs, 1606 tRNAs, 1236 rRNAs, and 1999 snRNAs were identified from *S. schlegelii* genome. 

### 3.3. Phylogenetic Tree Construction and Divergence Time Estimation

According to the results of genome comparison, 10,674 orthologous genes were shared among the genomes of *S. schlegelii*, *L. maculates*, *L. crocea*, and *C. semilaevis*. In addition, 52 gene families containing 353 genes were found to expand and 112 gene families representing 148 genes were found to contract in *S. schlegelii* genome when compared with those in *L. crocea* and *L. maculates* genomes. Meanwhile, we found that *S. schlegelii* possessed 551 specific genes, which were mainly involved in G-protein coupled receptor signaling pathway, transmembrane transport, and potassium channel activity, among others. The phylogenetic tree showed *M. musculus* located on the basal position of the tree as outgroup, and *S. schlegelii*, *L. maculates* and *L. crocea* were clustered together as a sister group to *C. semilaevis* and *P. olivaceus*. The divergence time analysis suggested that the initial appearance time of *S. schlegelii*, *L. maculates* and *L. crocea* was ~97.00 Ma (82.80–110.20 Ma) (Figure 1). 

### 3.4. Antimicrobial Peptides (AMPs) Identification 

**Figure 2:**
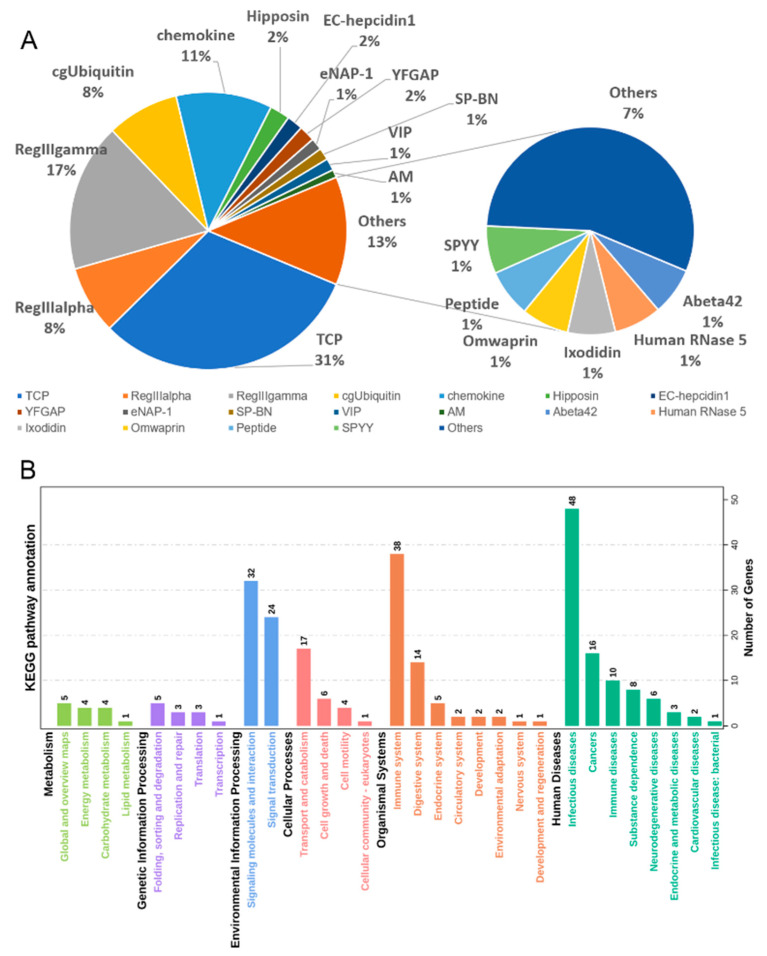
Identification and KEGG metabolic pathway annotation of AMPs in *Sebastes schlegelii.* (**A**) Classification of AMPs genes in *S. schlegelii;* (**B**) KEGG metabolic pathway annotation of 214 AMPs in *S. schlegelii*.

For the AMPs identification, we obtained 214 AMP genes in this genome (Table S5). Among these AMP genes, 213 were located on 24 chromosomes, from 4 to 24 on each chromosome (Figure S3). These AMPs can be divided into 33 classes, among which thrombin-derived C-terminal peptide (TCP) was the dominant type (31%), followed by RegIIIgamma (17%), chemokine (11%), and RegIIIalpha (8%), and cgUbiquitin (8%) (Figure 2A). To further explore the function of these AMP genes, we performed the KEGG pathway prediction for these genes. Totally, 101 AMP genes can be annotated in KEGG database, which participated in 94 KEGG metabolic pathways (Figure 2B). The results showed that 39 genes were involved in complement and coagulation cascades, chemokine signaling pathway, toll-like receptor signaling pathway, C-type lectin receptor signaling pathway, RIG-I-like receptor signaling pathway, cytosolic DNA-sensing pathway, IL-17 signaling pathway, Leukocyte transendothelial migration, intestinal immune network for IgA production, NOD-like receptor signaling pathway, which are all related to immune responses against infection. 

### 3.5. Comparison of AMP Genes in Teleost

For understanding the features of AMPs in teleost, we performed a comparison among those in *S. schlegelii* and other fish based on the known genomes and APD3 database. First, we predicted the AMP genes and then compared their numbers and types. The results showed that TCP, cgUbiquitin, RegIIIalpha, RegIIIgamma, chemokine, and hipposin are the top six AMP genes in these species (Table 2). In detail, the numbers of TCP were comparable among these species, while numbers of RegIIIalpha, RegIIIgamma, chemokine varied among these species. In addition, we found that *Oryzias latipes* harbors the largest number of antimicrobial peptides (817), followed by *Cyprinus carpio* (633), *Ictalurus punctatus* (547) (Table 2). Based on the phylogenetic tree, *I. punctatus* and *C. carpio* showed long distances with other species, and species such as *S. schlegelii* and *L. chalumnae* are newly evolved species. The numbers of AMP genes *S. schlegelii, L. chalumnae*, and *L. crocea* were 214, 281, and 504, respectively.

**Figure 3:**
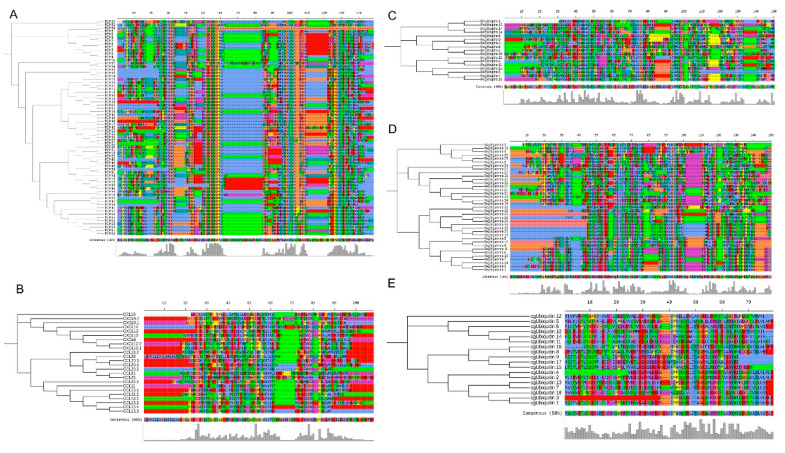
Alignment of AMPs in *Sebastes schlegelii* and their classical structures. (**A**) Alignment and classical structures of TCP genes in *S. schlegelii;* (**B**) Alignment and classical structures of chemokine in *S. schlegelii;* (**C**) Alignment and classical structures of RegIIIalpha genes in *S. schlegelii;* (**D**) Alignment and classical structures of RegIIIgamma genes in *S. schlegelii;* (**E**) Alignment and classical structures of cgUbiquitin genes in *S. schlegelii.* Different colors represent different amino acid sequences.

In addition, the top-five types of AMP genes from *S. schlegelii* were aligned to detect sequence diversity, respectively. In total, we identified 67 TCP in *S. schlegelii*, which shared 32.55% similarities (Figure 3A). Seven conserved amino acids regions were identified (PVC, ITMCAG, GGDS, CQGDSGGPLVC, GWLGVVSWG, GCA, KPGVYTRVSFDWI) in TCPs. Although these AMPs exhibited contractive gene copies, the results showed that the similarities of cgUbiquitin, RegIIIalpha, RegIIIgamma, chemokine were 42.63%, 29.87%, 28.09%, and 32.15% among the same types in *S. schlegelii*, respectively (Figure 3B–E).

### 3.6. Expression Patterns of AMP Genes in Healthy Tissues

**Figure 4:**
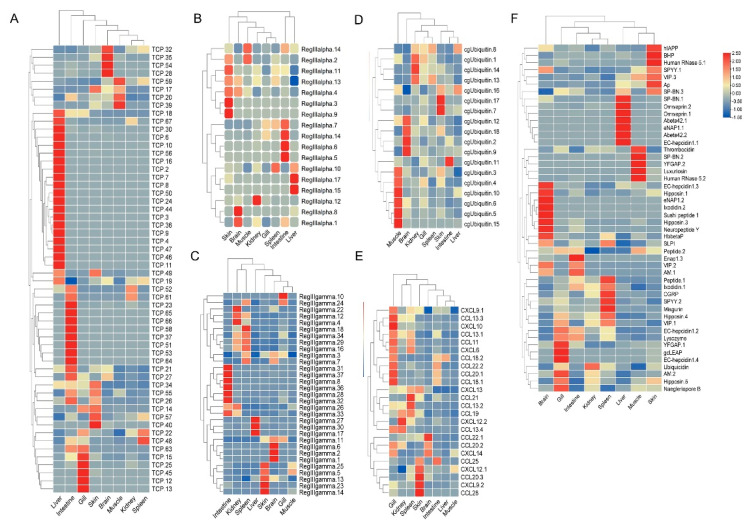
Expression patterns of AMP genes in *Sebastes schlegelii* among different tissues. (**A**) Expression patterns of TCP genes in *S. schlegelii* among different tissues; (**B**) Expression patterns of RegIIIalpha in *S. schlegelii* among different tissues; (**C**) Expression patterns of RegIIIgamma genes in *S. schlegelii* among different tissues; (**D**) Expression patterns of cgUbiquitin genes in *S. schlegelii* among different tissues; (**E**) Expression patterns of chemokine in *S. schlegelii* among different tissues; (**F**) Expression patterns of other genes in *S. schlegelii* among different tissues. Different colors indicate different expression values that were scaled to standard deviations. Red color indicates up-regulation and blue indicates down-regulation when compared to RPKMs of blood.

The tissue distribution patterns of these AMP genes in *S. schlegelii* were detected based on the transcriptome dataset. Overall, AMP genes were widely expressed in all the examined tissues (liver, spleen, intestine, gill, skin, brain, kidney, muscle, blood) with distinct expression patterns (Figure 4A–F). In detail, TCP showed higher expression levels in liver, intestine, gill, and skin. RegIIIalpha exhibited high expression levels in skin, intestine, and liver. Modest expression levels of RegIIIgamma were observed in the intestine, skin, and spleen. cgUbiquitin had relatively high expression levels in muscle, brain, and kidney. Chemokine showed high expression levels in gill, skin, and spleen. For other types of AMPs, we found that multi-copies showed distinct expression patterns. For example, higher expression level of hepcidin1.1 was observed in liver, while the higher expression levels of hepcidin1.2, hepcidin1.3, and hepcidin1.4 were found in the gill, brain, and gill, respectively. In addition, we also found that hipposin.1 and hipposin.3 both exhibited higher expression level in brain, while hipposin.4 and hipposin.5 exhibited higher expression levels in spleen and gill, respectively.

**Figure 5:**
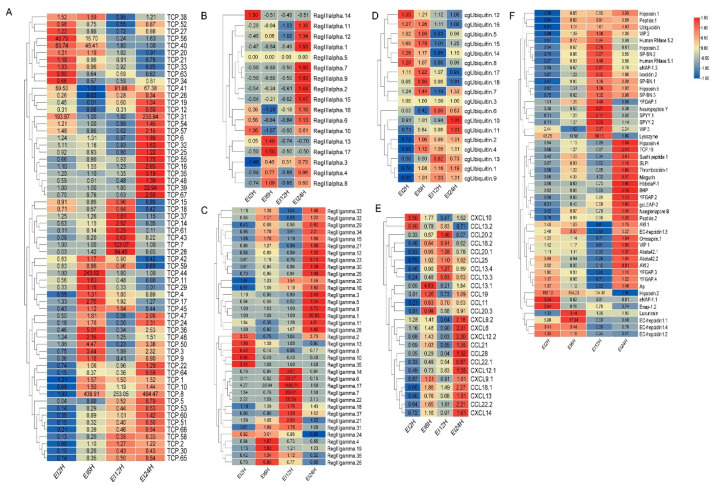
Expression patterns of AMP genes in *Sebastes schlegelii* after *E. tarda* infection. (**A**) Expression patterns of TCP genes in *Sebastes schlegelii* after *E. tarda* infection; (**B**) Expression patterns of RegIIIalpha in *Sebastes schlegelii* after *E. tarda* infection; (**C**) Expression patterns of RegIIIgamma genes in *Sebastes schlegelii* after *E. tarda* infection; (**D**) Expression patterns of cgUbiquitin genes in *Sebastes schlegelii* after *E. tarda* infection; (**E**) Expression patterns of chemokine in *Sebastes schlegelii* after *E. tarda* infection; (**F**) Expression patterns of other genes in *Sebastes schlegelii* after *E. tarda* infection. Different colors indicate different expression values scaled to standard deviations. Red color indicates up-regulation and blue indicates down-regulation when compared to the normal conditions. Values of relative fold change are marked on the heat maps.

The tissue distribution patterns of these AMP genes in *S. schlegelii* showed a high expression level in mucosal tissues. Therefore, we investigated the possible response patterns of AMP genes in *S. schlegelii* after *E. tarda* infection in intestine. The expression patterns of these AMP genes were detected based on the transcriptome dataset. Following the bacteria challenge, general expression patterns of some AMP genes showed up-regulation along with the infection time. Among the TCP types, TCP.56, TCP.40, YFGAP4, TCP.41 were up-regulated after 2 h infection (EI2H) compared to the control with 40.79-, 63.74-, and 65.95-fold, respectively. After 6 h infection (EI6H), TCP.8 and TCP.44 showed up-regulation with 439.91- and 243.02-fold, respectively. TCP.41, TCP.7, TCP.28, and TCP.8 were 91.88-, 123.07-, 88.45-, and 253.05-fold up-regulated after 12 h infection. After infection at 24 h, TCP.31, TCP.8 and TCP.39 were induced with higher expression levels (Figure 5A). Most of the RegIIIalpha and RegIIIgamma genes were induced at 24 h (Figure 5B,C). cgUbiquitin.7, cgUbiquitin.12, cgUbiquitin.15, and cgUbiquitin.16 were all induced following *E. tarda* infection at all time points (Figure 5D). We found most chemokines exhibited up-regulation at 6 h, 12 h and 24 h time points (Figure 5E). The expression patterns of other types of AMPs showed that two hepcidins were all induced at the initial infection stage (2 h and 6 h). In detail, hepcidin1.1 was 6.99-, and 17.34-fold up-regulated, respectively. After infection at 12 h (EI12H), a total of 15 AMP genes including lysozyme, VIP.3, and YFGAP.4 exhibited up-regulation (Figure 5F). Meanwhile, we found that more than half of AMPs were induced and in response to *E. tarda* infection at 24 h. Among these genes, genes such as Ixodidin.2, VIP.3, Hipposin.4, Thrombocidin-1, Misgurin, Hbbetap-1, AM.1, hepcidin.3, omwaprin.1, VIP.1, and luxuriosin exhibited more than 1.5-fold up-regulation following *E. tarda* infection. 

### 3.7. Phylogenetic Analysis of Hepcidins among Teleost

**Figure 6:**
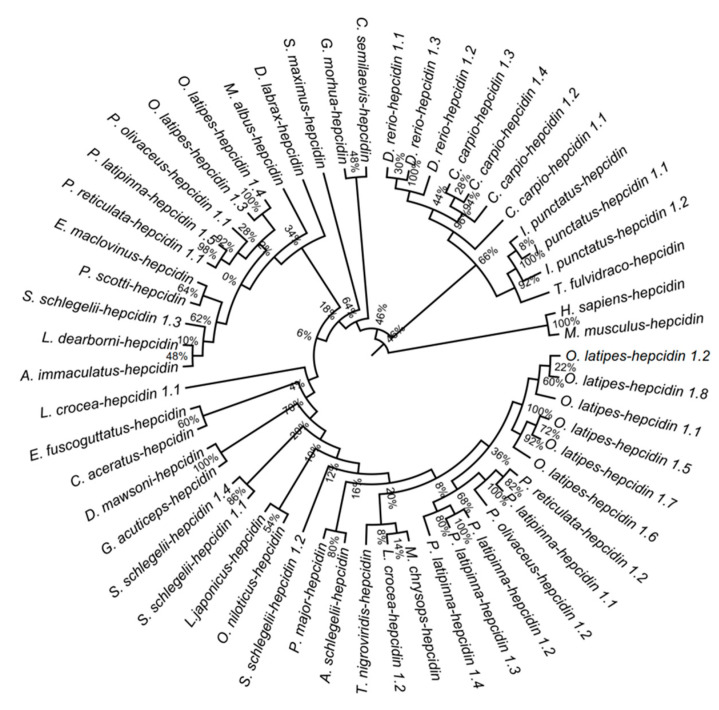
Phylogenetic of hepcidins among teleosts.

Hepcidin was originally identified in humans with direct antimicrobial activity towards pathogens [62,63]. Therefore, the sequence similarities and phylogenetic relationship of hepcidins among teleost were investigated. The results showed that hepcidins shared 70.06% similarities among 12 teleosts (Figure S4). The phylogenetic tree showed hepcidin1.1, hepcidin1.2, and hepcidin1.4 in *S. schlegelii* belong to the HAMP2 clade. *S. schlegelii* hepcidin1.3 belongs to the HAMP1 clade (Figure 6).

## 4. Discussion

A previous study proved that the evolution of species was determined by the variated genome sequences [64]. The interpretation of genomic information is an effective way to understand the characteristics of a species. Nowadays, the development of sequencing techniques and reductions in cost make the high-quality chromosome-level genomic sequences assembly for a species possible. The available genomes of fish provided valuable genetic resources for further investigation of evolutionary relationships and offered valuable database for breeding, selection of disease resistant strains, and characteristic analysis, among others [65,66,67]. *S. schlegelii* is an important mariculture fish in East Asian due to its high economic and ecological values [2]. In order to explore its genome features, we generated 24 pseudo-chromosomes for *S. schlegelii* using multiple sequence techniques, which is consistent with the karyotype of *n* = 24 in *S. schlegelii*. Our genome assembly, annotation, and phylogenetic evolution represent an important resource to further molecular breeding of this economically important fish, and also provide a database for the comprehensive understanding the molecular mechanisms of its immune responses. 

It appears that the antimicrobial peptides contribute to host defense independently and interact with adaptive immunity [68]. AMPs in teleost can be induced by various pathogens and have direct broad-spectrum antimicrobial activity towards both human and fish pathogens [32]. Despite a large number of reports on AMPs in humans, the identification and functional studies of fish AMPs still need to be further conducted. The first fish peptide was a toxic peptide that was discovered in *Pardachirus marmoratus* with antimicrobial activity in 1996 [69]. Since then, the number of identified and characterized fish peptides has progressively increased. To date, approximately 60 fish species have been reported to have their specific AMPs [35]. Genome-wide identification of AMPs has been applied to fish, suggesting their evolutionarily conserved characteristics. For example, over 400 putative AMPs were found in the blue tilapia and Nile tilapia [70]. In addition, a total of 254 putative AMP genes were identified in the giant grouper, *Epinephelus lanceolatus* [71]. In the previous study, only two hepcidin genes were identified in *S. schlegelii* [50]. In our study, 214 AMPs were identified in *S. schlegelii* genome, which is comparable with those in *E. lanceolatus* [71]. The sequence similarity analysis showed that these AMP genes were variated in their peptides, ranged from 22.15% to 63.85%. In addition, we performed a comparison of AMPs among *S. schlegelii* and other fish based on the known genomes and APD3 database for understanding the features of AMPs in teleost. The results showed that RegIIIalpha, RegIIIgamma, TCP, chemokine, cgUbiquitin, and Hipposin were the main components of the AMP genes in teleost. The dominate AMP types in the current study were TCP, accounting 31.3% of AMPs in this species. TCP was found to exert anti-inflammatory effects during the process of LPS-shock and *Pseudomonas aeruginosa* invasion [72]. We found 55 c-type lectin genes in *S. schlegelii* genome, which may act as a killer to microbes [73]. In total, 17 RegIIIalpha and 37 RegIIIgamma were identified in *S. schlegelii,* which were derived from regenerating islet-derived proteins and secreted C-type lectins, respectively. C-type lectin exhibited important roles in the immune response, such as pathogen recognition and cellular interaction [74]. We also found five histone-derived peptides, Hipposin, in *S. schlegelii*, which have broad spectrum anti-activities for Gram-positive and Gram-negative bacteria, parasite and fungi [75,76,77,78,79]. In addition, 16 CCL from chemokines were identified, which also have been demonstrated to play key roles in immediating immune response to microbes [80]. It has been reported that different copies of β-defensin genes were presented in fish [37,39]. Our study provided a systematic identification of AMPs in *S. schlegelii*.

For teleost fish, the mucosal surfaces (skin, gill, nose, and intestine) served as the first line of host defense against pathogens [81]. The tissue distribution patterns of these AMP genes in *S. schlegelii* showed high expression levels in mucosal immune system. Moreover, it has been proved that AMPs in teleost have the ability to eliminate the pathogens through their broad-spectrum antimicrobial activities [82,83,84]. It also has been proved that AMPs can shape the commensal microbiota and maintain the intestinal homeostasis [85]. What is the role of AMPs in intestine of *S. schlegelii* after bacteria invasion? The expression patterns of these AMP genes in *S. schlegelii* were detected in intestine after infected with *E. tarda*. The significant responsive patterns of these AMP genes in *S. schlegelii* indicated their important roles in response to pathogenic bacteria. Our results showed that most AMP genes were up-regulated after *E. tarda* infection. Results in our study showed ubiquitin was slightly up-regulated after infection with *E. tarda.* Previous study showed that ubiquitin played an essential role in modulating protein functions [86]. Moreover, we found that CCL18.1 exhibited down-regulation after infection at 6 h, 12 h and 24 h, which were generated from C-C motif chemokine, with key roles in immune response [38]. Interleukin-8 (IL-8) belonging to chemokine superfamily was originally discovered as a neutrophil chemoattractant in human [67]. IL-8 has been proposed to have a role in regulation of angiogenesis, cancers, and some other infections [87,88,89]. In our result, we noticed that IL-8 which contained CXCL6, exhibited significant up-regulation in *S. schlegelii* at different time points during challenge. Increased expression of IL-8 gene suggested that CXCL6 may function as a significant responsive factor during the process of pathogen infection. It has been proved that stimuli, such as bacteria, epidermal growth factor (EGF), and tumor necrosis factor (TNF), can induce the expression of IL-8 [90]. Previous study showed that IL-8 was induced in *P. olivaceous* after bacterial lipopolysaccharide (LPS) stimuli [91]. Similarly, it was found that IL-8 like genes in channel catfish and blue catfish were up-regulated after infection with *E. ictaluri* [92]. Histone-derived peptides can be produced in response to epidermal damage, LPS, or certain Gram-negative bacteria. In our study, we found that hipposin.2 was extremely induced by *E. tarda.* Moreover, an antimicrobial peptide hepcidin1.3 which is encoded by hepcidin-1 gene was also identified. The other three hepcidins were also induced when *S. schlegelii* was infected by *E. tarda.* The expression patterns of these AMPs may provide new insights and strategies for developing novel antimicrobial agents for *S. schlegelii* and can restore or even enhance the effectiveness of traditional antibiotics, thus increasing the disease resistance of *S. schlegelii*.

Hepcidin is a small cysteine-rich antimicrobial peptide and is also the key regulator of iron metabolism, which was originally isolated from human blood ultrafiltrate and named LEAP-1 [62]. Three domains—a 22–24 amino acid leader domain, a 40–47 amino acid proregion domain, and a 20–26 amino acid mature peptide domain—consistuted the fish hepcidins [93]. We totally identified four hepcidin genes in *S. schlegelii.* Sequences of similarities showed that hepcidins in *S. schlegelii* contained these conserved domains. Hepcidins in teleost have multi-copies. For example, there are 3, 4 hepcidins in *D. rerio* and *C. carpio*, respectively. Previous study showed that teleost hepcidins are classified into two clades (HAMP1 and HAMP2) [94]. Our phylogenetic analysis refined that four hepcidins in *S. schlegelii* can be divided into these two clades. To understand the ancestral origin and evolutionary history of hepcidin, more efforts should be conducted in future study.

## 5. Conclusions

In our study, multiple sequences techniques (shotgun sequencing, SMRT sequencing, 10× genomics, and Hi-C techniques) were used to assemble the *S. schlegelii* genome at the chromosome level, a species with high economic and ecological value which were widely cultured in East Asian countries. The genome features were analyzed including gene structures identification, protein-coding genes prediction and annotation combined with genome-wide phylogenetic relationship and divergence time estimation. Antimicrobial peptides are important molecules of innate immune system and exist in various organs and tissues of fish. Based on the whole genome database, we predicted 214 AMP genes and analyzed their features and gene expression levels in healthy tissues and after bacterial infection. Furthermore, a comparison of AMP genes among 12 fish showed that the number of AMPs genes contracted with the evolution. The whole genome of *S. schlegelii* provided a database for the comprehensive understanding of the AMPs, for which could in turn be beneficial for understanding the regulating innate and adaptive immune responses and could offer insights of accurate and effective design and development of AMP for aquaculture therapy in future. 

## Figures and Tables

**Table 1:** Statistics of the final assembly of Sebastes schlegelii genome.

Genome Assembly	Length		Number	
	Contig (bp)	Scaffold (bp)	Contig	Scaffold
Total	844,742,280	848,872,105	2113	744
Max	22,693,483	81,503,846	-	-
Number ≥ 2000	-	-	2081	714
N50	5,473,395	35,728,622	44	10
N60	4,291,763	33,987,958	62	13
N70	2,952,698	33,130,827	85	15
N80	1,543,679	28,667,948	123	18
N90	290,346	24,877,842	243	21

**Table 2:** Comparison the antimicrobial peptide (AMP) genes among *Sebastes schlegelii* and other fish.

AMPs	*Ictalurus* *punctatus*	*Cyprinus* *carpio*	*Danio* *rerio*	*Oryzias* *latipes*	*Poecilia* *reticulata*	*Poecilia* *latipinna*	*Monopterus* *albus*	*Paralichthys* *olivaceus*	*Cynoglossus* *semilaevis*	*Sebastes* *schlegelii*	*Larimichthys* *crocea*	*Latimeria* *chalumnae*
RegIIIalpha	57	58	31	121	72	79	23	18	22	17	150	7
RegIIIgamma	83	89	95	129	74	87	57	31	34	37	25	42
TCP	134	84	126	113	109	109	93	59	108	67	127	102
Chemokine	71	150	72	200	40	39	30	17	22	24	42	33
cgUbiquitin	36	71	30	37	31	29	27	13	26	18	46	27
Hipposin	30	28	41	23	15	17	19	9	28	5	26	13
Lysozyme	27	9	5	3	7	8	4	3	2	1	5	2
Histone H2B-1	20	1	17	0	0	0	4	0	3	0	0	0
HbbetaP-1	11	18	8	5	4	4	2	3	3	1	6	1
Vasoactive intestinal polypeptide	10	15	7	10	7	8	7	2	8	3	7	2
eNAP-1	7	12	10	39	5	6	5	3	3	3	4	2
BHP	7	2	1	2	9	7	4	0	1	1	0	1
Misgurin	6	0	4	6	0	4	0	0	5	0	5	0
SK84	5	1	1	0	2	2	1	1	2	0	1	1
Porcine NK-Lysin	4	8	5	2	0	0	2	1	0	0	2	0
Beta-amyloid peptide	4	4	2	10	3	4	4	2	11	2	4	1
Neuropeptide Y	3	1	1	5	2	2	2	2	1	1	5	2
Mouse Ang4	3	4	3	2	5	3	4	1	1	0	1	0
Adrenomedullin	3	7	3	10	2	2	2	2	3	2	6	2
YFGAP	2	3	2	2	3	4	2	3	2	4	1	1
SP-BN	2	9	5	4	5	6	4	3	4	3	6	3
Omwaprin	2	0	1	0	1	2	3	1	1	2	3	0
Human TC	2	1	1	5	2	3	0	1	0	0	0	4
HMGN	2	2	2	0	0	0	0	0	0	0	0	1
Hepcidin	2	4	3	8	2	5	1	2	1	4	2	1
gcLEAP	2	3	2	3	1	1	0	0	1	1	0	1
Calcitonin gene-related peptide	2	6	1	5	3	3	1	1	2	1	2	2
SLPI	2	11	2	0	0	0	2	0	1	1	1	1
Skin peptide tyrosine-tyrosine	2	4	3	5	2	2	4	0	2	2	0	0
Ubiquicidin	1	3	2	0	1	1	1	1	1	1	1	1
Thrombocidin	1	1	1	5	0	0	1	0	0	1	0	2
pCM19	1	2	1	0	1	1	1	0	2	0	1	1
Naegleriapore A	1	2	1	6	1	0	0	0	1	1	1	1
Human islet amyloid polypeptide	1	2	1	4	2	0	0	0	1	1	4	2
Ap	0	2	0	2	16	2	3	0	2	1	2	2
Peptide 3910	0	2	1	2	2	2	2	2	2	2	3	1
Sushi peptide	0	0	2	3	0	0	0	0	1	1	0	0
Luxuriosin	0	3	0	0	0	0	0	0	0	1	4	4
Buforin II	0	0	0	30	0	0	0	0	0	0	0	0
hPF4	0	0	0	6	0	0	0	0	0	0	0	0
BD	0	0	3	2	0	0	0	0	0	0	0	0
Ixodidin	0	3	1	0	0	0	0	0	1	2	2	1
gcLEAP	0	0	0	0	0	0	0	0	0	0	2	0
hGAPDH	0	0	0	0	0	0	0	0	0	0	3	1
Ceratoxin	0	0	0	0	0	0	0	0	0	0	2	0
Chemerin	0	0	0	0	0	0	0	0	0	0	0	3
Psoriasin	0	1	0	0	0	1	2	0	0	0	2	0
Chrombacin	0	0	0	0	0	0	0	0	0	0	0	5
Piscidin 4	0	0	0	0	2	1	1	0	0	0	0	0
Misgurin	0	1	0	0	2	0	0	0	0	1	0	0
Kaliocin-1	0	0	1	2	0	1	0	1	0	0	0	1
LEAP-2	0	1	1	3	1	0	0	0	1	0	0	1
Chicken LEAP-2	1	1	0	3	0	0	1	0	0	0	0	0
Amoebapore A	0	1	2	0	0	0	0	0	1	0	0	0
Fa-AMP2	0	3	0	0	0	0	0	0	0	0	0	0
Human Rnase	0	0	0	0	1	1	0	0	0	2	0	0
Enkelytin	0	0	0	0	0	0	0	0	0	0	0	1
Elafin	0	0	0	0	0	0	0	0	0	0	0	1
Total	547	633	501	817	435	446	319	182	310	214	504	281

## Data Availability

The data presented in this study are available in this article or Supplementary Materials.

## Supplementary Materials

The following materials are available online at https://www.mdpi.com/article/10.3390/biology10101015/s1, Figure S1: 17 k-mer frequency distribution of *Sebastes schlegelii*, Figure S2: Statistics of gene set evidences, Figure S3: The distribution of AMPs on chromosome of *Sebastes schlegelii*, Figure S4: Alignment of hepcidin in *Sebastes schlegelii* and other teleost, Table S1: Genome sequencing information of *Sebastes schlegelii*, Table S2: Chromosome length of *Sebastes schlegelii*, Table S3: Composition of repeat elements in genome of *Sebastes schlegelii*, Table S4: Gene annotation results of *Sebastes schlegelii* genome, Table S5: The AMP genes in *Sebastes schlegelii*.
