# Supplementary material for: Identification of Antimicrobial Peptide Genes in Black Rockfish Sebastes schlegelii and Their Responsive Mechanisms to Edwardsiella tarda Infection
Source: Biology (Basel). 2021 Oct 9;10(10):1015. doi: 10.3390/biology10101015 (PMC8533284; doi:10.3390/biology10101015)
Supplement: Supplementary file 1 [file biology-10-01015-s001.zip › biology-1334308-supplementary/Supporting Information/Table S1 Genome sequencing information of Sebastes schlegelii.pdf]

Table S1 Genome and transcriptome sequencing information of *Sebastes schlegelii*

Sequencing Platforms	Insert Size(bp)	Total Data（G）	Reads Length（bp）	Sequencing Coverage （x）
Illumina	350	102.30	150	121.32
Pacbio	-	84.18	-	99.83
10X Genomics	-	120.94	-	143.43
Hi-C	-	108.69	150	128.90
Total	-	416.11	-	493.48
