# Supplementary material for: Identification of Antimicrobial Peptide Genes in Black Rockfish Sebastes schlegelii and Their Responsive Mechanisms to Edwardsiella tarda Infection
Source: Biology (Basel). 2021 Oct 9;10(10):1015. doi: 10.3390/biology10101015 (PMC8533284; doi:10.3390/biology10101015)
Supplement: Supplementary file 1 [file biology-10-01015-s001.zip › biology-1334308-supplementary/Supporting Information/Table S2 Chromosome length of Sebastes schlegelii.pdf]

Table S2 Chromosome length of *Sebastes schlegelii*

Chromosome	Length (bp)
Chromosome1	81503846
Chromosome2	42791534
Chromosome3	42084525
Chromosome4	39370315
Chromosome5	38119448
Chromosome6	37751014
Chromosome7	36814648
Chromosome8	36511235
Chromosome9	36327873
Chromosome10	35728622
Chromosome11	35407004
Chromosome12	34988533
Chromosome13	33987958
Chromosome14	33795286
Chromosome15	33130827
Chromosome16	33111500
Chromosome17	30243101
Chromosome18	28667948
Chromosome19	28339358
Chromosome20	27944635
Chromosome21	24877842
Chromosome22	20741138
Chromosome23	17738797
Chromosome24	16978726
