# Supplementary material for: Identification of Antimicrobial Peptide Genes in Black Rockfish Sebastes schlegelii and Their Responsive Mechanisms to Edwardsiella tarda Infection
Source: Biology (Basel). 2021 Oct 9;10(10):1015. doi: 10.3390/biology10101015 (PMC8533284; doi:10.3390/biology10101015)
Supplement: Supplementary file 1 [file biology-10-01015-s001.zip › biology-1334308-supplementary/Supporting Information/Table S3 Composition of repeat elements in genome of Sebastes schlegelii.pdf]

Table S3 Composition of repeat elements in genome of *Sebastes schlegelii*

Repeat types	Type	Length (bp)	% in genome
Interspersed repeats	DNA	168,394,967	20.01
	LINE	87,329,927	10.29
	SINE	6,378,544	0.75
	LTR	67,572,128	7.96
	Other	71	0.00
Tandem repeats	Satellite	5,973,218	0.70
	Simple repeat	17,854,895	2.10
	Unknown	25,295,751	2.98
	Total	325,935,252	38.95
