# Supplementary material for: Identification of Antimicrobial Peptide Genes in Black Rockfish Sebastes schlegelii and Their Responsive Mechanisms to Edwardsiella tarda Infection
Source: Biology (Basel). 2021 Oct 9;10(10):1015. doi: 10.3390/biology10101015 (PMC8533284; doi:10.3390/biology10101015)
Supplement: Supplementary file 1 [file biology-10-01015-s001.zip › biology-1334308-supplementary/Supporting Information/Table S4 Gene annotation results of Sebastes schlegelii genome.pdf]

Table S4 Gene annotation results of *Sebastes schlegelii* genome

Database	Annotation	Percentage (%)
Swissprot	21,614	89.60
Nr	23,001	95.30
KEGG	19,833	82.20
InterPro	23,942	99.20
GO	22,390	92.80
Pfam	19,660	81.50
Unannotated	124	0.50
Annotated	24,010	99.50
Total	24,134	NA
