# Supplementary material for: Identification of Antimicrobial Peptide Genes in Black Rockfish Sebastes schlegelii and Their Responsive Mechanisms to Edwardsiella tarda Infection
Source: Biology (Basel). 2021 Oct 9;10(10):1015. doi: 10.3390/biology10101015 (PMC8533284; doi:10.3390/biology10101015)
Supplement: Supplementary file 1 [file biology-10-01015-s001.zip › biology-1334308-supplementary/Supporting Information/Table S5 The AMP genes in Sebastes schlegelii.pdf]

Table S5 The AMP genes in *Sebastes schlegelii*

>evm.model.Chr15.1148-Peptide.1
RADTQTYQPYNKDWIKEKIYVLLRRQAQQAAK
>evm.model.Chr17.652-Peptide.2
RADTQTYQPYNKDWIKEKIYVLLRRQAQQAAK
>evm.model.Chr16.786-eNAP-1.1
SIKCPDGNVCSDRATCCRTSHGYSCCPYPNAVCCSDLAHCCPSGY
>evm.model.Chr18.174-eNAP-1.2
DVPCDGTGESHCSVQETCCKVSATEWACCPYARAVCCSDSKHCCPVGY
>evm.model.Chr14.847-eNAP-1.3
NVTCDDTHACPDDTTCCKTTTGDWACCPLPKAVCCDDHEHCCPEG
>evm.model.Chr14.1459-SPYY.1
YPPKPENPGDDAPPEELAKYYTALRHYINLITRQRY
>evm.model.Chr18.809-SPYY.2
YPPKPENPGGNASPEDWAKYHAAVRHYVNLITRQRY
>evm.model.Chr6.452-Hipposin.1
GKDSGKAKAKAVSRSQRAGLQFPVGRIHRHLKTRTTSHGRVGATAAVY
>evm.model.Chr9.122-Hipposin.2
SGRGKKPVPPPKTSVSRSTRAGVTFPVARIHRLLKKGQYSRRVGTGAAVYF
>evm.model.Chr20.125-Hipposin.3
GKDSGKTKTKAISRSQRAGLQFPVGRIHRHLKSRTTSHGRVGATAAVY
>evm.model.Chr12.430-Hipposin.4
GGKKKATKLSRSARAGVIFPVGRMMRYLRTGTHKYRIGMGAPVYM
>evm.model.Chr10.326-Hipposin.5
GAKKKSTKTSRSTKAGVIFPVGRMLRYIKRGLPKYRIGVGAPVYL
>evm.model.Chr5.1004-Misgurin
RKRVEELSKFSKKGAAARRRK
>evm.model.Chr3.506-Luxuriosin
CSLPAVQGPCKSWEPRWAWNSLLKQCQAFVYGGCHGNANSFRTKKECE
>evm.model.Chr22.802-Ixodidin.1
RCPPNSHYKTCGSACPPSCEYNATFCTKICVQGCFCNPSFIRSQIGCVRPHQC
>evm.model.Chr24.305-Ixodidin.2
YKVCGSACPETCGLQPEACSKICVEGCFCDPGYVLSGQECVIREKGC
>evm.model.Chr18.977-BHP
FASYPGTKTYFSHLDISPRSAHLLSHGKK
>evm.model.Chr15.851-Naegleriapore B
CWACKWALKKVKKDLGNNATVEKLKSKLMSICDQIGLLKSLCRKFVKVHLP
ELIEELTTT
DDVKTICVNTGACKPKDMS
>evm.model.Chr8.871-CXCL14
KCRCTRKGPKIRYKDVQKLEIKPKHPFCQEKMIFVTMENVARFKGQEYCLHP
KLQSTKNL
VKWFRIWKDKHR

>evm.model.Chr3.1119-Thrombocidin-1
RCRCISKETRFIRLRNIEEVEVNPPSSHCNAIEIIALKKGGQRICLHPDARWVKK
LLEMR
>evm.model.Chr16.815-Neuropeptide Y
YPVKPENPGDDAPAEDLAKYYSALRHYINLITRQRY
>evm.model.Chr2.555-CGRP
ACNTATCVTHRLADFLSRSGGLGHSNFVPTNVGAQAF
>evm.model.Chr12.18_evm.model.Chr12.19-VIP.1
HSDAIFTDNYSRFRKQMAVKKYLNSVL
>evm.model.Chr19.608-VIP.2
HSDGIFTDSYSRYRKQMAVKKYLAAVL
>evm.model.Chr11.335-VIP.3
HSDGIFTDSYSRYRKQMAVQKYLAAVL
>evm.model.Chr2.529-AM.1
KRSKNSANQSRRQGCSLGTCAVHDLAHRLHQLNNNLKIGTAPIDKIRPQGY
>evm.model.Chr5.280.1-AM.2
GCALFTCAYHDLLHKLHQLTNKEKELNAPTIHISSKGY
>evm.model.Chr5.510-Ap
YTPAQDGPYTVAVKYADQEVPHSPFKVMSQPGHDASKVRASGPG
>evm.model.Chr1.398-SP-BN.1
ATGDVCQDCTQIFELLADLLSNADLQKKIMDGIENVCDHLPGPASAKLCKEEV
EKMLPVA
IHFIVGVVKPAEVCKLIGLC
>evm.model.Chr12.874.1-SP-BN.2
CDLCKEVLIVVEQILKDNATEAEVLGYLEKACQLIPDAGLTAECKEMVDSYYP
IIMGIIK
GELEDPGVVCGALGLC
>evm.model.Chr6.1157-SP-BN.3
DMCSECSQVIQLSANMISSRDTKETVYEALHALCQRLPGERASECDSQVKMY
LPKVLQQT
PGHLKPGETCMVFGLC
>evm.model.Chr23.48-TCP.1
HLPIVEPNICRSSTSVRTTDNMFCAGYKPEDAHRGDACEGDSGGPFVMKYPA
ENRWYQMG
IVSWGEGCDRDGKYGFYTHVFRMSRWMRKIIDAAGK
>evm.model.Chr23.32-TCP.2
HLPIVEPNICRSSTSVRTTDNMFCAGYKPEDAHRGDACEGDSGGPFVMKYPA
ENRWYQMG
IVSWGEGCDRDGKYGFYTHVFRMSRWMRKIIDAAGK
>evm.model.Chr23.37-TCP.3
MPYVNRQTCIESTQMRISTRMFCAGYDTIAKDSCQGDSGGPHVTRYHNTYFIT
GIVSWGE
GCARKGKYGIYTQVSKYIRWIHDGIEQL
>evm.model.Chr10.407-TCP.4

VPHVDRTLCKQSSRDHVTRYMFCAGFRNEKKDSCQGDSGGPHSSNYKGTWF
LTGIVSWGE
ECAMDGKYGIYTRVSRYYPWISQ
>evm.model.Chr8.395-TCP.5
VPFTDRTECKRSSSARITPVMFCAGYYDEAKDACQGDSGGPHTNRLHDTWFL
TGIVSWGE
ECAKQGKYGVYTRLSLYYRWINHVM
>evm.model.fra_scaf239un.2-TCP.6
TLPVVSYRDCTASTVQVITDNMFCAGYLDVGMDACSGDSGGPFVVNYRGTW
FLTGVVSWG
EKCAAKGKYGVYTRLGNFLNWIRDTMET
>evm.model.Chr11.1312-TCP.7
VPLVDRGVCSRQMSHNISENVLCAGVLGQRMDACEGDSGGPMVTLYRDTWF
LIGLVSWGE
GCGREDKLGIYTKVSNYNEWIGRV
>evm.model.ori_scaf515un.3-TCP.8
VPLVDRGVCSRQMSHNISENVLCAGVLGQRMDACEGDSGGPMVTLYRDTWF
LIGLVSWGE
GCGREDKLGIYTKVSNYNEWIGRV
>evm.model.Chr23.39-TCP.9
VPRIRTQQCLEESHVALTENMFCAGYIEGRQDSCKGDSGGPLVTEYKKKAFLL
GIVSWGK
GCARPGNYGIYTRVSNYLEWIH
>evm.model.Chr3.1428-TCP.10
LVSEEACVRTYGHLVTPRMLCAGYRSGDKDACQGDSGGPLVCQEPSGRWFL
AGVVSWGRG
CGRPDYYGVYTRITRLTDWITQVI
>evm.model.Chr23.40.1-TCP.11
LPRVPQQECRLHTKLNITRNMLCAGLKTGGQDACRGDSGGPLVTRYKKTWFL
TGVVSWGK
GCANENLYGVYTRVSNFLDWIQDIM
>evm.model.Chr14.953-TCP.12
VPIVGNRECNCNYGVGSITDNMICAGLRTGGRDSCQGDSGGPMVSKQDGRWI
QSGVVSFG
EGCARPNRPGVYTRVSRYMAWINSEI
>evm.model.Chr20.903-TCP.13
NVPIVGNNECRCAHRRLTDNMICAGLRAGGKDACTGDSGAPLVTKKGGVWI
QSGIVSFGN
GCARPNNPGGYTRVSQYQEWITSI
>evm.model.Chr18.160-TCP.14
ISDNMFCAGRPDWSQDACEGDSGGPLVCEVGSRLFLFGVISWGDGCAKENRP
GVYTRVTN
YNRWIEE
>evm.model.Chr22.810-TCP.15

VKIINDTVCNVVTEGQVTSRMLCSGFLAGGVDACQGDSGGPLVCFEESGKWF
QAGIVSWG
EGCARRNKPGVYSRVTKLRDWIKK
>evm.model.Chr24.519.1-TCP.16
HVPIISDAVCNAPDYYDNQITTSMFCAGYEKGGTDACQGDSGGPFVAEDCLS
KTRRYRLL
GVVSWGIGCAMAKKPGVYTRVSRFLPWISTAM
>evm.model.Chr11.813-TCP.17
VPLLPQSTCKSALGKELVTNTMLCAGYLSGGIDSCQGDSGGPLIYQDRISGRF
QLYGITS
WGDGCGEKGKPGVYTRVSAFSDWIQSEIQKSFG
>evm.model.Chr9.162-TCP.18
SIPLVDRAKCSSPTIYGSAITQRMICAGLLEGGVDACQGDSGGPLVYFTSAKW
HLVGVVS
WGVGCARKGRPGVYCNVEELLNWIHTVIEK
>evm.model.Chr1.2162-TCP.19
VPIVSTARCNSSESFNGNITANMICAGYRTGGKDACKGDSGGPLVCRGRVYGV
VSWGNGC
GDPKFPGVYTAVSRFRRWIDQTI
>evm.model.Chr12.294-TCP.20
VPLLPAWKCKKQYSDRFTSRMLCAGSLSEHRRVDSCQGDSGGPLVCQGEGGR
WVLTGVIS
WGHGCGNPLFPGVYTRVSRFLRWIDKVINK
>evm.model.Chr19.465-TCP.21
VSSRMLCAGVPSGERDACRGDSGGPLSCQAPGGGRWFLIGIVSWGAGCGRPN
LPGVYTRV
NKFTSWI
>evm.model.Chr12.858-TCP.22
ITENMFCAGSPDWSTDACKGDSGGPLVCEASGRMFLFGVVSWGDGCASKNK
PGVYTQVTN
YNKWI
>evm.model.Chr22.977.1-TCP.23
IIAQPVCSEPSIYGTFLTPRMICAGTMEGGVDSCQGDSGGPLVCETASGDWKL
AGVVSWG
EGCGRRNKPGVYSRVTQLIHWVKR
>evm.model.Chr23.38-TCP.24
SVPIVQNSQCSQRAQFNFTDNMLCAGYLEGRQESCRGDDGSPLVTLYGSTHFL
TGVVGWG
RGCAHPGYYGVYANMANFVDWVEGVM
>evm.model.Chr9.160-TCP.25
VTKNMLCAGHLNGGKDSCQGDSGGPLVCEKDKRWYLAGITSWGEGCGERN
RPGVYTDVKS
VLPWIYSSMQQ
>evm.model.Chr16.315-TCP.26

DLPILPQLDCERSYPNRITSSMFCAGFLEGGKDSCQGDSGGPLVCNGELQGVV
SWGWGCA
EENRPGVYAKVCHFTDWIHSTM
>evm.model.Chr19.466-TCP.27
SVKIINRNACNKLYDEAVTARMLCAGNLQGGVDACQGDSGGPLVCLERGRR
WFLAGIVSW
GEGCARQNRPGVYTQVVKFTDWIHQ
>evm.model.Chr21.90-TCP.28
NVTILPPDVCNQFYRGRMRPSMFCAGKDGGGADACQGDSGGPLSCFTGSRY
ELAGLVSWG
IGCGRATRPGVYTNVQHQTQWMSDIM
>evm.model.Chr4.609-TCP.29
IIDSKVCNKSSVYRGAVSHNMMCAGFLQGQVDSCQGDSGGPLVCEGAPGRFF
LAGVVSWG
VGCAMINRPGVYSRVTRLRNWILSYAD
>evm.model.Chr24.61-TCP.30
PLLSDDTCFNSYPFQITENMICAGYLEGGKDSCQGDSGGPMTCDGELQGVVS
WGHGCALR
KKPGVYTKVCNYISWIKNTM
>evm.model.Chr7.191-TCP.31
LPIVSTVKCNSSESYNGNITENMICAGFSTGGKDACQGDSGGPLVCDGRVYGL
VSWGVGC
ADAQFPGVYTAVSKFRRWIDNTIFSY
>evm.model.Chr6.265-TCP.32
ITSNMLCAGDTRGHDDACKGDSGGPLVCRNNDKMTLMGVISWGDGCGQKD
KPGVYTRVTH
YIDWINSKI
>evm.model.Chr8.237.1-TCP.33
IINSNVCNDLMGGGITSRMLCAGVLTGGVDACQGDSGGPLSSSSGTRMFLAG
VVSWGDGC
ARRNKPGIYTTVTKFRGWVKE
>evm.model.Chr18.663-TCP.34
VPIISQSSCQEMYRTDPKEQVDILYDMICAGYQQGGKDSCQGDSGGPLVCQTV
NGTWVQA
GVVSFGLGCAHANKPGVYARLTSYTSFITNTVPEL
>evm.model.Chr3.1038-TCP.35
LSLLPRRHCQQHFHSAFTSRMLCAGSIQPERRVDSCRGDSGGPLVCERPGGG
WVVYGVTS
WGHACRTQQSPGVYTKVSAFSSWIRKVI
>evm.model.Chr17.703-TCP.36
FPVIENKICNRPSYLNGRVKDHEMCAGNIEGGTDSCQGDSGGPLVCNSQNRY
VLQGVTSW
GLGCANAMKPGVYARVSKFVDWIDTTI
>evm.model.Chr3.672-TCP.37

LPVVDFQTCSKPAYWWDTLRPSMICAGYGSPDELKSACQGDSGGPFACVAAG
MNTTWEVH
GVVSFGPQGCIKDKKPSVFTRVSAFSDWISNNIKKF
>evm.model.Chr18.556-TCP.38
LDDSMFCAGNMKGGVDSCQGDSGGPLVCERNGTHYVVGVVSWGDGCGKK
YKPGVYANVGR
FVDWI
>evm.model.Chr13.952-TCP.39
ISSWMICAGYLEGGIDSCQGDSGGPLACEDSSGWKLVGATSWGEGCAMRNK
PGVYTRVTQ
ALSWIHQQMENL
>evm.model.Chr7.190-TCP.40
PYCNYYYWGRITPNMLCAGSRYGGKDSCQGDSGGPLICSGKFEGIVSWGISC
ANPYFPGV
YTKVRMYVSWINWIIDH
>evm.model.Chr18.288-TCP.41
LPIIDHKTCRQKKFWGDRVRDSMICAGIRDTEGPPAACQGDSGGPLLCQQGQ
DRWEVHGV
VSFGPIGCTVENKPSVFTRTRTYIPWIE
>evm.model.Chr5.1292-TCP.42
VLRTVNRRPARPQPAMTVLCAGPERGGKDACQGDSGGPLVCPAGSGGGHWV
ALGVTSWGK
GCGRSWGNNGGRSPSRRGSPGVFTDVRRLLPWIK
>evm.model.Chr16.525-TCP.43
PIIDDRICRNAYPHIFTQNMVCSGFMHGGASSCQGDSGGPLVCNGQLQGVVS
WGYDCAMK
GHPSVYARVCRYNSWI
>evm.model.Chr20.867-TCP.44
CQNFYKGRFKPGMMCAGDLEGSVDSCQGDSGGPLVCEDELGVSYLWGIVSW
GERCGQSGF
PGVYTQVAHYFEWIR
>evm.model.Chr18.657-TCP.45
IPVVGQNQCSCNYRPVTNVNITDEMVCAGRDNKGACQGDSGGPLQCKQGPR
WIQSGITSF
GVPCALPGFPGVYARLSEFQTWI
>evm.model.Chr20.868-TCP.46
IKPGMICAGDLNGRVDACEGDNGGPLVCEDELGVSYLWGIVSWGRGCGRPRS
PGVYTQVM
AVSCRTKKPVMSHFG
>evm.model.Chr12.975.1-TCP.47
LDNSMFCAGHLQGGVDSCQGDSGGPLTCEQNSTSVIYGLVSWGDQCGKKDK
PGVYTQVTH
FLDWIKSKIQTF
>evm.model.Chr20.923-TCP.48

HVSADMICAGLRGCSDACQGDSGGPLACARGDVSFLYGIISWGDGCGHSKKP
GVYTKVVN
YIDWINSVI
>evm.model.Chr1.2161-TCP.49
RITDNMICAGSRLGGKDSCQGDSGGPLICNGRLEGVVSWGIGCAYAYYPGVY
TKVRNYLD
WMK
>evm.model.Chr18.557-TCP.50
RLDSSMICAGTLQGGIDSCQGDSGGPLVCERNGTNYVTGVVSWGLGCAERN
KPGVYANVH
AFSSWIKSKMN
>evm.model.Chr22.821-TCP.51
LPLLSPAQCKQYWGSNRISDAMICAGASGVSSCQGDSGGPLVCENSGVWSLV
GIVSWGTS
NCNVRTPAVYARVSYLRSWIDQIV
>evm.model.Chr10.745-TCP.52
ITETMICAGKLQGGVDSCQGDSGGPLVVKQGDVWWLAGDSSWGIGCAWRN
NPGVYGNVTY
FIDWVYR
>evm.model.Chr11.475-TCP.53
LPVVDHTICSQSDWWGSSAKTTMVCAGGESKSACHGDSGGPLNCEGRDGK
WYVQGVTSFV
DGRGCNTPRKPTVFTRVATFIPWISETMVQ
>evm.model.Chr18.366-TCP.54
LCAGSPPDTSLLHDDSCQGNSGGGLVCQGEMGRWVLTGVVAGGYGCASPSSP
ALYTRVSR
FRSWIEEVTN
>evm.model.Chr14.1512-TCP.55
ISIIPHNVCKAKYPTLTSDQLCAGGVEAGGEDACQGDYGGPLMCRAADGGDV
QVGIMSYG
SPAGCGLPGRPGVYTKVFKYLSFIDAYTKQ
>evm.model.Chr2.617-TCP.56
LPLANHSVCKAEYERNPFTPAVDDNMFCTGPTKYDENVCFGDAGGALAVTD
AETGDIYAA
GILSFDKSCVSHKYGVYMKICSYLPWIHSVI
>evm.model.Chr5.419 1013-TCP.57
EQQYARNGVPVSVTDNMLCASHKPDYEPSNICPSDTGGILVLPALTENQASND
QKASLRV
TQAESKGLWRLLGLVSFGYDQGECDPDLYTVYTRVANFKDWIE
> evm.model.Chr1.1593-TCP.58
LPVVDHKTCSSYGWWGSTVKDSMVCGGGGRESGCQGDSGGPLNCSVNGK
WVVHGVTSFVS
SSGCNANKKPTVFTRVSAYISWMNGIM
>evm.model.fra_scaf239un.3-TCP.59

LPVVSYRDCTASTVQVITDNMFCAGYLDVGMDACSGDSGG
>evm.model.Chr4.985-TCP.60
LPVVGHSVCSSSSWWGGTVKPTMICGGGDIRSGCHGDSGGPLNCRGVDGRW
YYVQGVTSFV
SSRGCNTLRKPTVFTRTSSFTKWISDTM
>evm.model.Chr4.54-TCP.61
LPVVEHSVCSRSDWWGINAKSTMICAGGDAVSGCNGDSGGPLNCVGQDGRW
YYVQGVTSFV
SSRVCNEVKKPTVFTRTSAFTDWLSDV
> evm.model.Chr9.398-TCP.62
RISQDMLCARETEEVAHMCHTDSGGPLVSLKDGVWWLVGDTIWGEHCTEQN
KPGVYGNVT
YFLDWI
> evm.model.Chr14.113-TCP.63
SVPIVTNNRCSEAYTSITSNMGDSGGPLVSKMGSIWVLGGVVSFGRGCAEPNF
PGVYARV
SEYQSWI
> evm.model.Chr19.532-TCP.64
IPVVEHSVCSQPGWWGSIALRTMVCAGGDGVISGCQGDSGGPLSCFTDGAWR
VHGVVSYG
PSGMCNQVSKPTVFTRVSSFTDWIYSVV
> evm.model.Chr6.712-TCP.65
MPVADHATCSKSDWWGIAVRTTMVCAGGDGIVAGCNGDSGGPLNCKNTEGS
WEVHGIASF
VSGLGCNYVKKPTVFTRVSAFNDWIDQVM
> evm.model.Chr7.1241-TCP.66
LPVVDHATCTKPDWWGPQVKDTMVCAGGDGVVSGCNGDSGGPLNCQKTD
GAWEVHGIVSF
GSGLSCNFPKKPTVFTRVTSYIDWISSTMVAY
> evm.model.Chr15.1216-TCP.67
VVSSARCRRSDYFGRGKFTNNMICANRICSHHCDQPTEDTCDGDSGGPLLYN
GIAVGITS
NGGKKCGQAKKPGIYTVISHYTAWIDNIM
> evm.model.Chr4.241-Omwaprin.1
RSSRQGDCPPPQRATGFAAACVESCSSDQHCPSPRKCCSNGCGHTCQ
>evm.model.Chr23.688-Omwaprin.2
KQGRCPPPERASGFEAACVESCDHDRECSGPKKCCSNGCGHTCQSP
>evm.model.Chr18.976-HbbetaP-1
AAKMGTSFKAETQAAFQKFLTVVVAALGRQYH
>evm.model.Chr10.938-Sushi peptide 1
GYTMRGSSKRVCLPNGKWSGFTPICSRD
>evm.model.Chr20.618-gcLEAP-2
MTPLWRIMNSKPFGAYCQNNYECSTGICRAGHCSTSHRSPTEPVN
>evm.model.Chr13.411-Abeta42.1

DTEERQSAGYEVYHQKLVFFAEDVGSNKGAIIGLMVGGVVIA
>evm.model.Chr23.490-Abeta42.2
ETEDRQSTEYEVHHQKLVFFAEDVGSNKGAIIGLMVGGVVIA
>evm.model.Chr24.468-EC-hepcidin1.1
MKTFSVAVAVAVVLAVICIQESSALPATKVQELEEPMSNDNPVAADHEETSVDS
LKMLYN
NREKRDLKCSFCCNCCITGCGVCC
>evm.model.Chr24.469-EC-hepcidin1.2
MKTFSVAVAVAVVLIFICFQESSAFPFAGVKELDEPMSNDEPAAENEEMPVSSR
KMPFNI
RQKR
>evm.model.Chr22.967-EC-hepcidin1.3
MKAFSIAVAVTLVLAFICILESSAVPFTGVQELEEAGSNDTPVAAYQEMSMESR
MMPNHI
RQKRQSHLSLCRWCCNCCNRYKGCGFCCKF
>evm.model.Chr24.467-EC-hepcidin1.4
MKTFSVAVAVAVVLAVICIQESSAVPATKVQELEEPMSNDNPVAADHEETSVD
>evm.model.Chr22.456-YFGAP.1
VKVGINGFGRIGRLVTRAAFTSKQVEIVAIND
>evm.model.Chr16.1066-YFGAP.2
VKIGVNGFGRIGRLVTRAAATGGKVEVVAIND
>evm.model.Chr24.51-YFGAP.3
VGINGFGRIGRLVLRACLQKGIKVVAIND
>evm.model.Chr24.524-YFGAP.4
VGINGFGRIGRLVLRACLQKGIKVVAIND
>evm.model.Chr12.325-cgUbiquitin.1
MQIFVKTLTGKTITLEVEPSDTIENVKAKIQDKEGIPPDQQRLIFAGKQLEDGRT
LSDYN
IQKESTLHLVLRLR
>evm.model.Chr11.641-cgUbiquitin.2
MLIKVKTLTGKEIEIDIEPTDKVERIKERVEEKEGIPPQQQRLIYSGKQMNDEKT
AADYK
IQGGSVLHLVLALR
>evm.model.Chr11.927-cgUbiquitin.3
GIPPDQQRLIFAGKQLEDGRTLSDYNIQKESTLHLVLRLR
>evm.model.Chr20.639-cgUbiquitin.4
VERIKERVEEKEGIPPQQQRLIYSGKQMNDEKTAADYKIQGGSVLHLVLALR
>evm.model.Chr12.542-cgUbiquitin.5
MELFIETLTGTCFELRVLPFEAVISVKAKIQRLEGIPVAQQHLIWNNLELDDEHC
LHDYG
IAEGCTLKLVLAMR
>evm.model.Chr6.629-cgUbiquitin.6
MILTVKPLQGKECSVQVTEDEKVSTVKELVSERLNIPANQQRLLYKGKALADE
HRLSDYS

IGPEAKLNLVIR
>evm.model.Chr1.897-cgUbiquitin.7
MWIQVRTIDGKETRTVEDLSRLTKIESLRLKIQEIFNVSPPQQRLFYRGKQMED
GQTLFD
YNVGLNDIVQLLIR
>evm.model.Chr22.79-cgUbiquitin.8
EVTVKTLDSQSRTYTVGAQLTVKEFKEHIAPSVGIPVDKQRLIYQGRVLQDER
TLADYNV
DGKVIHLVER
>evm.model.Chr14.1502-cgUbiquitin.9
MQITLKTLQQQTLQIQIDPEQTVKALKEKIEAERGKDNFPVSGQKLIYAGKILQ
DDTPIK
DYNI
>evm.model.Chr10.414-cgUbiquitin.10
QLRLRLSTGRDLRLAVRSSDTVGMMKRRLHSQEGVPAATQRWFFSGRPLTDR
LRLDQLNI
SRDYVVQVIL
>evm.model.Chr12.489-cgUbiquitin.11
QLRVRLSTGKDVRLTASMADSIAELKKQLKEQEEIEVTRQRWFFSGKLLTDKT
RLQDAKI
QKDFVVQVIVNI
>evm.model.Chr12.1215.cgUbiquitin.12
MIVFVRFNQGPGVAVELQEESSVAELKEVVGRQQGVPAELLRVLFAGRELRSN
STLQGCD
LPEQSTVHVVL
>evm.model.Chr4.118-cgUbiquitin.13
MWIQVRTMDGKETHRVDSLSKLTKVDELRLKILELFKIEPERQRLFYRGKQM
EDGHTIFD
YNVGLNDIVQLLVR
>evm.model.Chr18.1039_evm.model.Chr18.1040-cgUbiquitin.14
FQLKVRLSTGTDLRLSASMADTIGLLKKQLQAQEDIDANHQRWFFSGKLLTD
KTRLQDTK
IQKDFVIQVIV
>evm.model.Chr4.1108-cgUbiquitin.15
LTITLKTLQQQTFKIEIDPELVVAALKEKIEKDRGKDAFPAAGQKLIYAGKILND
DTPLK
DYKIDEKN
>evm.model.Chr6.573-cgUbiquitin.16
LNGQVLNFTVPLTDQVSVIKVKIHEATGMPAGKQKLQYEGIFIKDSNSLAYYN
MNNGSVI
HLALKER
>evm.model.Chr1.1001-cgUbiquitin.17
MQITLKTLQQQTFKIDIDEEETVKTLKEKIEEEKGKDHFSVAGLKLIYAGKVLS
DDAALK

EYKI
>evm.model.Chr22.66-cgUbiquitin.18
MVVNVKTPNGKEEISIPEDASVSQFKQEVSKKFEAQQDQLVLIFAGKILKDGDTLKQHGI
KDGLTVHLVIK
>evm.model.Chr8.857-Human RNase 5.1
QYQKFIKQHINEKMSVDMCDSVMQARKIAKIKNKCKQINTFILSNIRTVKSICEDKGEAY
GDMTKSFERFDIVVCKLEKRQTWPVKCHYRGEQKLKKKIIIKCEGDLPVHYERDI
>evm.model.Chr22.569-Human RNase 5.2
NNGYNTFIKRHIRSGTPDTLDQNLWEAYIKNNGGCDRPTQSFLHQRDLDRVKAVCTNQGG
VVYKENLCISRQPFTFVTVRSVPGTCGIKSVREETKHLILACEELSNQCLPVHFE
>evm.model.Chr4.423-RegIIIgamma.1
CEHTWRKFHGHCYRYFSRRHTWEDAEKDCREHSGHLASIHSLAEQNFIRGLSHDNTWIGL
NDRTVEDDFQWTDKMDLQYENWRENQPDNFFAGGEDCVVMIAHENGKWNDVPCNYNLPYV
CK
>evm.model.Chr11.838-RegIIIgamma.2
GDTCEKDVEGCEHGWRKFHGHCYRYFTHRHTWEDAEKDCREHSAHLSSVISATEQEFING
LGHDNAWIGLNDRTVEEDFQWTDSNDLVYENWRESQPDNFFAGGEDCVVTIAHEDGKWND
VPCNYNLPYICK
>evm.model.Chr20.113-RegIIIgamma.3
APTDQLVSAQMCPQGWTFHGGHCYTLSTEHKVTWSTANRACRERYKGTLASVLSKVDMDW
LWDFSGRKPFWIGLNDRDGRGRWEWAGGEPVSYTNWRKTPPRS
>evm.model.Chr11.328-RegIIIgamma.4
FKGYCYAFFTDSRQWADASVECGKHGGSLASIEDPSEQEFIQSNVKTFEDSHNSFWIGLF
QTHKGEWLWLDKTVMDFTNWAEGLPDHRSYGGISASDGTWTTGSNWYHRPYICK
>evm.model.Chr24.114-RegIIIgamma.5
KEQQALQTVCLRGTKILGKCFLADPVKKTFHAASEDCIAKGGSLATSLSGDENDQLYSYV
RQSIGPEEHIWLGINDIVTDGQWVDQSGASVRFKNWETEITLQPDGGRSQNCGILSTTAN
GKWFDESCRAEKASVCEF
>evm.model.Chr22.163-RegIIIgamma.6
CEPGWEKFQSFCYRHFAKRQSWEAAEQHCRMCGGHLLSVMTPEEQDYINDK

YREYQWIGL
NDRTIEGDFRWSDGNPLLYENWYRGQPDSYFLSGEDCAVMVWHDGGRWSD
VPCNYHLSYT
CK
>evm.model.Chr5.230-RegIIIgamma.7
DCSTPVQGCAEGWLEFMGSCYLHFAERDTWSEAEQRCQELNAHLVSIGSQEE
QQFVNSNG
QDYQWIGLNDKDVQNEFRWTDSSPLTFENWRPNQPDNYFNSEEDCVVMIWH
EGGQWNDVP
CNYHLPFTCK
>evm.model.Chr1.582-RegIIIgamma.8
FNDYCYLFNYLSMRTWAEARADCVNQGGDLVSITDPFEQAFIQGVIQLSPTGI
SLWMGGH
DSVTEGGWEWADGSPFRYIRWAAGNPDNYYGEDCLSILINNGYWNDDNCEY
NRGYICKRK
G
>evm.model.Chr13.875-RegIIIgamma.9
KDETTIPVSCPDHQKAFGGSCYEFVDLQRTFFSGQAWCEQRGGHLAFIPDEET
QYFLQRL
LDPKKDAWLGLAPSASPKPRHSLSVEGALSWLDGSHITYSNWVSSPQPGAAC
GHILRDSD
FQWKATRDCNEKLHFICQFDS
>evm.model.Chr2.391-RegIIIgamma.10
CEDGWTKFQGNCYLNFADREMWLEAELRCRDLNAHLVSIITPEEQNFVNSNA
QDYQWIGL
TDKTVEHDFRWTDGTPRQYENWKPDQPDNYFHSGEDCVVMIWHENGQWN
DVPCNYHLPFT
CK
>evm.model.Chr16.533-RegIIIgamma.11
CETGWDKFHGFCYRHFSQRLSWEVAEQHCRTQGAHLVSIITPEEQSYINDNYK
EYQWTGL
NDKTIEDDFRWSDGNPLLYENWYRGQPDSYFLSGEDCVVMVWHDDGRWSD
VPCNYHLAYT
CK
>evm.model.Chr11.327-RegIIIgamma.12
FKGYCYLFITEEIEWADAASSCIRHGGSLASIADPAEQQFIKSYVVIFQDSLSSF
WIGLY
KTHKGSWLWLDKTVMDYTNWAEDEPVNDFGGIGTSDGAWRTGRRWHDRA
YICK
>evm.model.Chr2.509-RegIIIgamma.13
SCPKHQEGFDGSCYEFVGLQRSVLSAQGWCERGGGHLAFILNDEKQQFLQK
HLDPEKDWW
LGLAPAAPNLTLDSAATEASLAWLDGSDVSYSNWVNMPEAQAACGHILRHSG
FQWEAAGN

CSKELDFICQFDS
>evm.model.Chr6.773-RegIIIgamma.14
TCEYGWHKFQGHCYKYFPHRRNWDTAERECRIQGAHLTSIITHEEQQFVNRL
GQDYQWIG
LNDKMFDSDFRWTDGRHVQYENWRPNQPDSFFSSGEDCVVMIWHEDGQWN
DVPCNYHLTF
TCK
>evm.model.Chr2.228-RegIIIgamma.15
VLISDSKDWWSARNHCREFHGDLVTITNLEEATELVPYEGWIGLRRVKTGGW
RWSRGDEK
VNYVNWDDGEPDRNEHCVWKRQSHLKWINHPCNERLPFIC
>evm.model.Chr3.197-RegIIIgamma.16
CPTGWVMFQTSCYFTAIGKSTWNESRKYCQSKSADLAIVTTAEEMAFINGLYS
IDKEVWI
GLTDGGIEGQWKWVDETALTTPFWAKGQPNSFDGRNQDCVEFWHRATGRGE
WNDEHCGIE
QHFICE
>evm.model.Chr6.494-RegIIIgamma.17
EVLLPKLVPVRGSCREGWVSFERSCYLLSTSAVIWRNAEEQCRTRGGHLAVIN
NVEELDY
ISKIVEIQYNYWIGLVERQHEGHWSWVDGTDFNSTPTFWDEGQPDNWDYRE
NGEDCGQLH
ASQIQKRKMWNDADCNLRYRYICETRA
>evm.model.Chr13.1057-RegIIIgamma.18
CPAGWRGFSCACYLLSEESGSWEKGREDCKDREADLVVIGSSEEQMFLSNFA
ETGTQAWI
GLTDRVEERTWMWIDGAALSLKYWLKTQPDNGGGHGTLGEEDCAHIITGGN
NFNNWNDLS
CNTRMRWICE
>evm.model.Chr20.64-RegIIIgamma.19
KTWTEAASYCRRTYTDLASIENTEEMNQLMNTVSSSSNSSEVWIGLYSAIVW
KWSDGYRG
TGSEYRNWETSVNEPNFHSGGQLCVFTAGYGRWFDEVCNYEYPFIC
>evm.model.Chr20.66_evm.model.Chr20.67-RegIIIgamma.20
KTWTEAASYCRRTYTDLASIENTEEMNQLMNTVSSSSNSSEVWIGLYSAIVW
KWSDGYRG
TGSEYRNWETSVNEPNFHSGGQLCVFTAGYGRWFDEVCNYEYPFIC
>evm.model.Chr20.399_evm.model.Chr20.400-RegIIIgamma.21
ERILRNSSTVEGCCPLDWDKLGSSCYLFSKTALSWDEARDWCNGHESHLVIL
NTDEEWEF
VISHIMGKFYWVGLTDERTGEWEWVNQTPYVMNRRRWMPGQPDSWTGHGL
GRGDEDCAHL
HSQGRLNDLHCSTKMRYICQ
>evm.model.Chr5.819_evm.model.Chr5.820-RegIIIgamma.22

AQCPPDWLADGRSCYTVRATGLTWSDAQHSCRGLAAGSHLADLKTVEDLLF
LSSHLLSHN
NLLLLWTGLNDQQEEGRPLWSDGSASNSTNTMMSLLPANQTDCFALQRNAT
GPGYFLTPF
FCNIPLPFIC
>evm.model.Chr16.439-RegIIIgamma.23
KERQALQTICLKGVKIHDKCFLADTVRKRYHAASEDCNNMGGVLGTPTSSD
GNDQLRDYV
RQSIGPDEQVWLGINDMVTEGTWVDQTGSSITYKNWDTSNSRSPQPDGGQSH
DCAVLSGA
SQGKWFDENCREEKASVCQF
>evm.model.Chr2.712-RegIIIgamma.24
ALEPEPNPNATCPDGWMSKPKVKYCYKVFHEERLSRKRSWEEAERFCQALG
ANLPSFTTN
AEMRDLHSIMRNTISDNRYFWVGLNRRNPADRSWQWSDGQPVPLDVLQQDF
HEDDAFSRN
CAAFKSSRTSLKHLFSFLLHDLP
>evm.model.Chr17.727-RegIIIgamma.25
CQKGHRMGYKCYLVYNSQEDYAGAARKCLERGGRMAMPRDRREQENLAD
YVKSFFHPGNW
PVWLGVNDLRSEGLYLFDDGSRVSYFQWRKHFLSSQPDGGRRENCVAMSSD
DGDWWDHYC
DRTMNYLCEF
>evm.model.Chr20.61-RegIIIgamma.26
NWSSAQRYCRENYTDLATVRNDTETQEIDNLVWRGNYAWIGLFRDPNFNWS
DGSNYKFSY
WDSAKNPLGSLTHVCGVALQSSGKWRAFDCERRLPFVC
>evm.model.Chr22.424-RegIIIgamma.27
SECPEGWLHVGDQCYHFSNDKLDWLQSRDSCAKMGSHLTILHTMEQHDALE
IEAKKIGGF
DYHFWIGLSDIEKEGEWRWVDNTTLKHKYWDPWSSEPNNHQSGGEHGEDC
ATLD
>evm.model.Chr20.93-RegIIIgamma.28
FVVVNERMNWSSAQRYCRENYTDLATVRNDTENQEIQSLVPSGNYAWIGLFR
DPNLNWSD
GSNYKFSYWDNALNPLSSLTHVCSAALQSSGRWRAFDCERRLPFVC
>evm.model.Chr15.587-RegIIIgamma.29
CPSGWIHLNSSCYFFSSTESSTTKKNWYDSRADCIRRGADLVVIDNQEEQTFV
SLTIEHM
EDGYVWKGYWIGLTDTEIEGQWVWINNVTEVEQKYWIDGEPNNQGYQGED
CG
>evm.model.Chr16.1040-RegIIIgamma.30
CPVDWHLFNDHCYFISRTTRDWPESQSYCQSKGAYLAIIHTAEEQTFLWDLLP
RGHWNAF

WFGISDEHTEDQWKWVDGTPLVGGFWEVGEPNNHINEDCGYIVKTQALERV
AIRSWYDAP
CTMYWPFICE
>evm.model.Chr3.1309-RegIIIgamma.31
KNLTKERDECKKLAEKPSCLCPWITFGYSCYLVSTSKKNWHDSRTFCKQQDA
DLVIISSL
QEQEFISSLNQNCWIGLIDEAIISPVWKWVDGTAVTIEYWRMGQPDNHNNLEQ
LCSVFIL
WCDTY
>evm.model.Chr9.300-RegIIIgamma.32
KSWIYALDHCYGRISSLVQITNITVWNAVKSLLQNKTELQKGVWIGLERSIFG
KPDRPWK
WISGSIAKDDEPPWNSSLVDPLNNHCGKIIRDKNSQELKLLDSNCDDKLPFICQ
GK
>evm.model.Chr20.68-RegIIIgamma.33
KTWTEAASYCRRTYTDLASIENTEEMNQLKNTVSSSSHSSEVWIGLYMAIVW
KWSDGYRW
TGSEYRNWGTTYNNPNFLSGREFCVVASKSGEWHDNMCDSGRPFIC
>evm.model.Chr16.1018-RegIIIgamma.34
SCEEGWEQHGTKCYHFSSKKSTWEESRRECKGLGGDLVKIESREKQKFLLRR
LRDKMDSH
EDKFWIGLTDSEEENKWLWADGSPLNTSLMFWSFKEPDNWDGENKKDGEDC
VRMGEKNGA
HLMWWFDKDCRVPHRHIC
>evm.model.Chr20.581-RegIIIgamma.35
NWTEAQRFCRENYVDLASIRNWTDNDIITNLAGGDFVWIGLHREKLWSDGSP
SLFRHWAN
EQPSSGAEECVTTSFNDSGRWSDDNCFLRLPFIC
>evm.model.Chr20.60-RegIIIgamma.36
KTWTEAASYCRRTYTDLASIENTEEMNQLKNTVSSSSNSSDVWIGLYSAIVWR
WSDGYRG
TGSRYRNWQTAYSEPNFKSGDEFCVTTGFNGLWWDFSCNAKYPFIC
>evm.model.Chr16.1074-RegIIIgamma.37
SPCPAGWTPQGEKCFLFSQDRADWISSQYRCMALGAAVATVRTEEEQVFLWQ
KAQSLSQG
DSYWLGLRSSAGGDGGWRWSDGSPVEKGTGFWESEPDKTDSGGELCGRLTP
GDDYRKSWF
TYRCSNLLRSICE
>evm.model.Chr20.773-RegIIIalpha.10
CPHEWSRFGNQCFVFIDNPKTWSEAESYCLFEVANLASVMSPEEEHFIQALTR
GDTHDFP
QVWIGGHDAVHSCFWMWSDGSKFSYENWAKDYNVERNEHCLMMNYGHHR
KWNYASCDDTL
PFVC

>evm.model.Chr14.618-RegIIIalpha.11
CYQFNFQATLSWSEARISCQQQGADLLSITKLHEQTYINGLLTGYSAALWIGL
NDLDING
GWQWADSSPLKYLNWESEQPNHAEEENCAVIRTESSGRWQNRDCTVALPYV
CK
>evm.model.Chr19.600-RegIIIalpha.12
PSKDQPSFCPSHWVPYAGNCYSLQRSKKMWKDALAACRKEGGDLASIHNIEE
QSFVISQS
GYLPTDVLWIGLNDRRNQMLFEWSDHSHVTFTQWQSNEPSHATNLQEDCVLI
RGKDGKWA
DHMCEKTLGYICRKT
>evm.model.ori_scaf1087un.1-RegIIIalpha.13
CGFGWQKFQSHCYKYFTHRRTWDAAERECRLHGAHLASILSQEEQQFVNRL
GSDYQWLGL
NDKMFERDFRWTDGNPMQYDHWRPQPARQLLPVRRGLCGDDLARGRPVER
RACNYHLTFT
CK
>evm.model.Chr3.1310-RegIIIalpha.14
SSSQQCPAGWRLINCRCYFVSTEDKTWMDSRKYCQSKDADLVVINSEEEQKS
LYRLNGDD
GLMYWIGLHVPTGSTGWKWVDGCALTKSFWTPGQPNRNPMKVEDCVTMRF
SYPELANWHD
YPCSAKQRWLCE
>evm.model.Chr6.53-RegIIIalpha.15
HGFVATPMTWAAAEKHCQTMGANLASVHSKDEHQYLLQFTKQNNVNQETW
VGGSDCQEEG
QWLWSDGSAFEHTEWCERKPDDENHVLNCLQMNHGVDFCWDDYKCSATRP
FVC
>evm.model.Chr10.399-RegIIIalpha.16
EQPAAKAPPAEKDALCHQEGCYTVFLQKRTFREAGRSCRERGGTLATMHTQE
AAGVVHEL
LSAIEGTRSRLRLWIGLHRPPRQCSSTRSLRGFVWVTGDQDGQFTNWLREDTP
GTCAVPR
CVAMTVHTSESGRESSDNFRWVDGSCALPLDGYVCQY
>evm.model.Chr22.425-RegIIIalpha.17
QCPEGWLVVGDQCFLLTTDRDDYSNSTNKCAEIGAHLAILTTKEQHDAVEKE
GKNIGGIY
TYYWIGLTDIETEGDWRWVDNSKLRTPFWEAPEPNNHLSGGPEGEDCAVVQS
YTQLWHDV
PCSFTYPRICQM
>evm.model.Chr20.34.1-RegIIIalpha.1
WSDALFFCRRHYWDLLSLRSQEEQSEVEQLLSGSPFPLTDYVWLGLRRSLMG
ESWFWMSG
DSMEFTKWPRDFAPHHYSNPCGGMAKGGLSLWEDQPCEESLNFICQ

>evm.model.Chr17.82-RegIIIalpha.2
PDSGYCIGTRCFTVFTEHSDFTTARDQCTARGGHLMTVRSTVSNDVLQILLGN
VMGRFWI
GLHLQTGCPDPAAELRGFQWVTGDQETDFSKWPQSFNSSCSPPPPRCVSVSSE
DEF
>evm.model.Chr2.348-RegIIIalpha.3
SFDDAEKACNQHTSGIYVGHLVSIHSLDELCQVECAMYNEQNGKAHYWIGL
RRTKPMEDD
DDADSNLAWTDATESHYFRWAGGQPDNHDGEENCIEMNYGEWGLWNDEDC
LQKKPYVC
>evm.model.Chr2.713-RegIIIalpha.4
CYQFNLYTILTWSQALSTCQAQGGNLLSITNLAEHRYIRDRLASVGVMVWIGL
NHLKDGR
GWQWSDGAPLSLVNFTTGLPASPLKDNRRCGVYNSAYEGHWQSLTCESALPY
ICKKT
>evm.model.Chr20.732_evm.model.Chr20.731-RegIIIalpha.5
CPPRWLLFGQRCFSFYLVWSSWTNANSLCSQEGGNLASLHAPEERQFVSQLA
NTHTPVWL
GGFKAQQKGSWFWSDDSAFRISGW
>evm.model.Chr20.1003-RegIIIalpha.6
NWSSAQRYCRENFTDLATVRNDAENQEIKSLVWSGDRAWIGLFRHPNLTWSD
GSNYKFSY
WDYLNPIGSLVHVCGVADLQKSGKWRGYNCEMRLPFVC
>evm.model.Chr20.758-RegIIIalpha.7
CDGGWSEFHGRCFYYVPIVMTWAQAEKNCLSMGGNLASVHNAMEYHEIQR
LILMSSHEYK
QAWIGGSDAQEEKQWFWIDGTPFNYMNWCAGEPNNSRGKQNCLHMNHAAP
KCWDDLQCHN
RVPSVC
>evm.model.Chr10.939-RegIIIalpha.8
KGRPCYKLAYFSELRRRLNFVEAELACRRDGGQLLSVESASEQKIVEQLITEL
RPTDGDF
WIGLRRNHGNEDSSSDCSSQYYWLDGSKSTFRNWHWDEPSCGYEVCVVMY
HQPSAPPGLG
GLYMFQWNDDNCETKNNFICKYT
>evm.model.Chr8.1223-RegIIIalpha.9
KGWFQMGDRCLKAFYHNKHPNFQDAENICRKYGGNLVSIHNIVELCQVECI
MWRTSRDKA
HYWIGLHKTPERRLHYTWTDGSGNTDGWTHWAWGQPDIMPREHCIEMNYW
DWGLWNNVDC
MKGRPYVCAM
>evm.model.Chr11.893-CCL20.1
CCTRYNRKPVPFQRIKGFREQTTKENCHIEAIILYTVKKNEICATRRDEWVRKV
LELLSS

KLKKM
>evm.model.Chr19.739-CCL20.2
SCCMRYTNRRFKCQRLMGYTIQTINTSCDINAVIFHLPGRFVCADPLTKWTQ
>evm.model.Chr4.534-CCL20.3
DVQCCMLYSQGKVRTKDVLRFEVQTEGPDCSIQAIILYTKKAVKCADPRDRK
VKRLLRKL
LQR
>evm.model.Chr12.470-CXCL9.1
GRCLCPQTQPGVRGQLKELSVYPKSPSCDKVTVIVTLKSNNEPVCLDPGAPM
GRQLTRCW
>evm.model.Chr3.109-CXCL9.2
GRCSCLNTIKFVKGNMSDFQVLEKRPGCDKTELIVTMNNPDNSTEQFCMNNE
GKMAKAFL
KCWE
>evm.model.Chr3.1120-CXCL10
LGVELHCRCIQTESKPISRHIEKVELIPANSHCGETEIIATLKKTGQEVCLDPEAL
WVKK
VIKRIMSNR
>evm.model.Chr3.551-CXCL12.1
KPISLVERCWCRSTLNTVPQRSIRELKFLHTPNCPFQVIAKLKNNREVCINPET
KWLQQY
LKNAINK
>evm.model.Chr12.473-CXCL12.2
KPISLVERCYCRSTVNNVPRVYIRELRFMHTPNCPFQVIAKLKSNKEVCVNPE
VRWLQQY
LKNALNR
>evm.model.Chr6.168-CXCL13
CRCIRTIPNAIPRRIIRKIEVIPISGHCRRTEIIITRRNGSTVCVDPSAVWVEALLR
NLQ
NENGNS
>evm.model.Chr11.895-CCL11
SCCRKLSKTQIHRDLLKSYYKQDTQSCPIYAVVFTTLKGIRICGDPDRVWTKTS
MAYLDG
K
>evm.model.Chr20.274-CCL13.1
PDNCCFEHYARRLDKRLVRSYIMTDRRCPKTAVILLTKRSRRICVDPNRSWVK
SIMGMVD
KR
>evm.model.Chr5.1367-CCL13.2
PKRCCFRFNENEVPKERVVGYIKTSQRCSNPAVLLKTVAGRQLCARPSDAWV
GKLISYLD
AKANPGQT
>evm.model.Chr17.393-CCL13.3
PDKCCWSFYSSRLPMNRVVSYKYTDNRCSMEGVVMKMKKGHEICVDPSEL

WV
>evm.model.Chr17.392-CCL13.4
PDKCCATFISSRLRTNNVMSYKYTDNSCPMEGVIFKRINGNELCADPSQLWVK
>evm.model.Chr5.1395-CCL18.1
CCFQFFTGQVPQKQIISVVKTHSNCHEKGFVVSTARRKEICVSQNLNWAQK
>evm.model.Chr11.819-CCL18.2
ATAYHGCCRSYMTGKIPFAKIKGYSVQDVTQLCHIPAIIFHTPRGRACTNPAHN
WVMVYI
NLLR
>evm.model.Chr6.205-CCL19
EDCCLVVSTKPVPLKLIANYIILEAGNGCDISATVFITKAGRKLCVSHPDDRPW
VKTGAE
KKTRLSQSAFQR
>evm.model.Chr11.274-CCL25
QTTYEDCCFKYVKRMNHRTQRHAVKYRLQVTDGGCNIPAVIFTMKRGRDLC
TDPREDWVA
ELKQKIDAKAEMKVDARSGNTGRRKFKK
>evm.model.Chr4.538-CCL21
AQGSFGNCCLGYVPRMNARVKANVESYIEQKTDGQCNIPAIVFLIKRKPSHTR
QHTRCAN
QEHIWVQELMRAVD
>evm.model.Chr2.412-CCL22.1
GPVAQTAVNSVCCPGYTKTRIPLARVKHIGMTGSNCASRAIVFTTVCNRAHCI
DPDLDWA
KDQLKKFNE
>evm.model.Chr5.1394-CCL22.2
SMAPGNCCFKVSTSSLPLRLVSDITKTHSSCPKKAFIVQTIKGRKICYSGTFPW
ALDVYN
Q
>evm.model.Chr7.1212-SLPI
KPGVCPRRNWGSGACAEFCSNDSDCPNDEKCCHNGCGHSCIEPYTVKRGRC
ALPKGTLMC
AEYCYHDGQCPGEQKCCKTTCGHACSEP
>evm.model.Chr17.337-Ubiquicidin
KVHGSLARAGKVRGQTPKVDKQEKKKKKTGRAKRRIQYNRRFVNVVPTFG
KKKGPNANS
>evm.model.Chr20.670-CXCL6
HCRCLQVESRIIPPDSLKSIKLVPEGPHCPDIEVIAGLTNGEKVCLNPRSSWVKK
LIQFV
LE
>evm.model.Chr6.898-CCL28
LKVVLLIVCLAALAIYSTEAGIPKCCITTKKVVPDSVLLKVQRMSVQQSNGAC
DIPALIL
YVNNTRRPICAHPKVKKRLRALQWMK

>evm.model.Chr21.750-hIAPP
KCNTATCVTQRLADFLVRSSNTIGTVYSPTNVGSGTY
>evm.model.Chr3.578-Lysozyme
YGLFQLSDATFCDSGYRRSKNRCHTDCRAFTDDNIMDDIDCIVRTGYWRRIQ
KKCRRVNA
AEYFAKC
