# Supplementary figures and images for: Identification of Antimicrobial Peptide Genes in Black Rockfish Sebastes schlegelii and Their Responsive Mechanisms to Edwardsiella tarda Infection
Source: Biology (Basel). 2021 Oct 9;10(10):1015. doi: 10.3390/biology10101015 (PMC8533284; doi:10.3390/biology10101015)

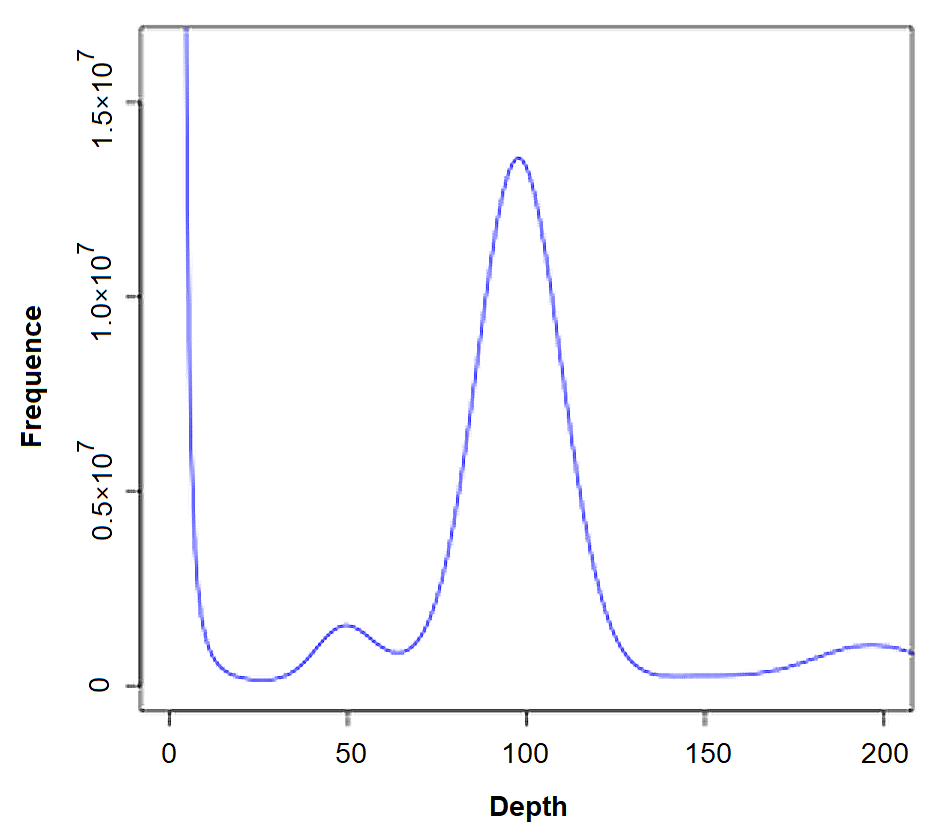

Supplement: Supplementary file 1 [file biology-10-01015-s001.zip › biology-1334308-supplementary/Supporting Information/Figure S1 17k-mer frequency distribution of Sebastes schlegelii.tif]

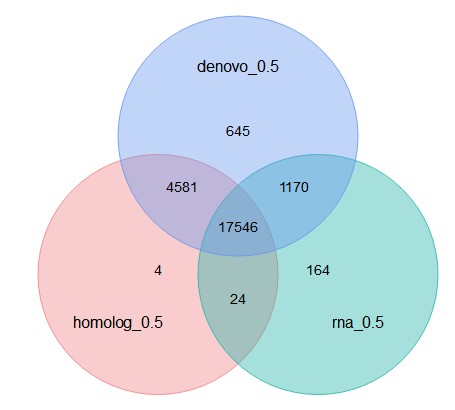

Supplement: Supplementary file 1 [file biology-10-01015-s001.zip › biology-1334308-supplementary/Supporting Information/Figure S2 Statistics of gene set evidences.jpg]

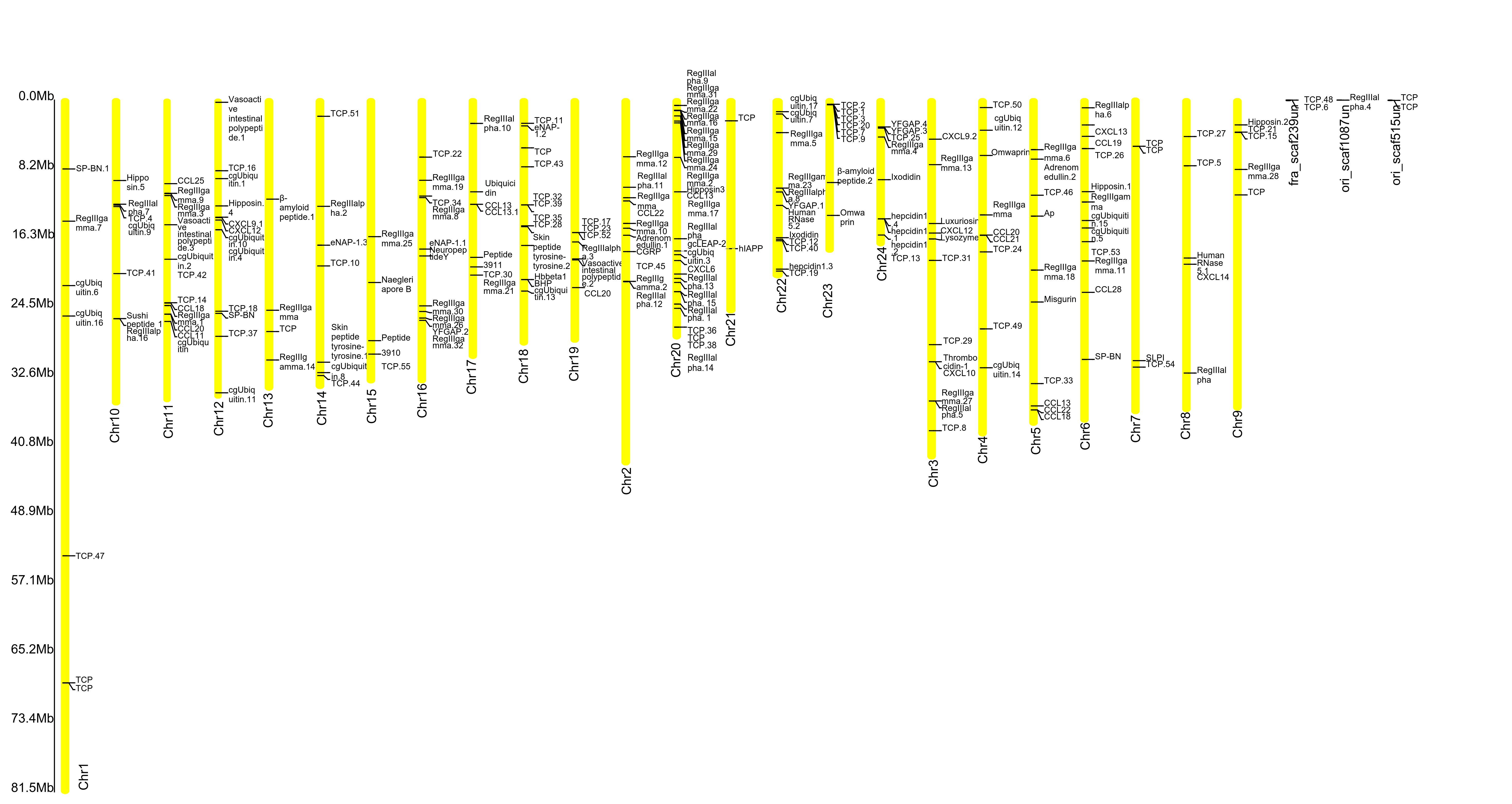

Supplement: Supplementary file 1 [file biology-10-01015-s001.zip › biology-1334308-supplementary/Supporting Information/Figure S3 The distribution of AMPs on chromosome of Sebastes schlegelii.tif]

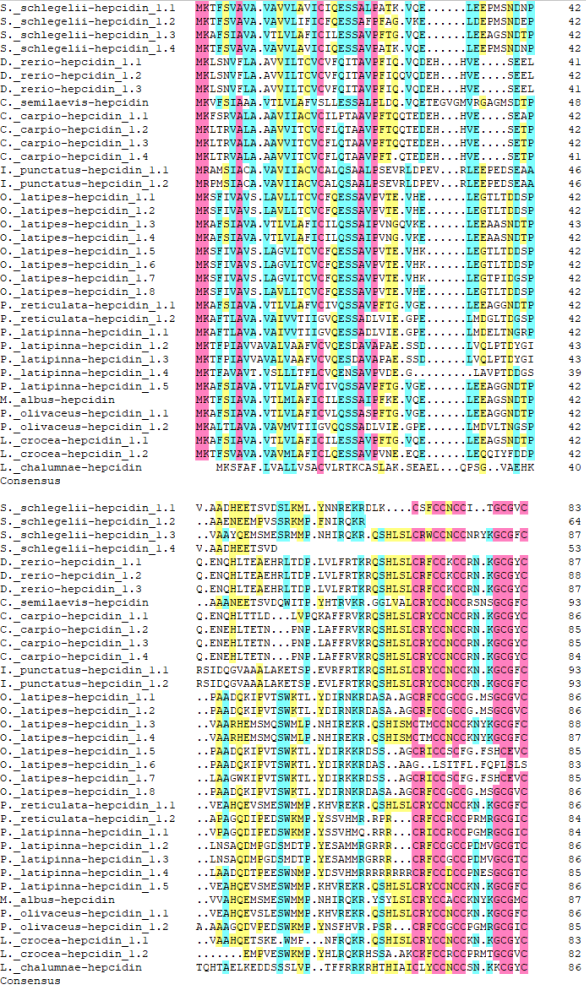

Supplement: Supplementary file 1 [file biology-10-01015-s001.zip › biology-1334308-supplementary/Supporting Information/Figure S4 Alignment of hepcidin in Sebastes schlegelii and other teleost.png]
